# Evidence for Within-Host Genetic Recombination among the Human Pegiviral Strains in HIV Infected Subjects

**DOI:** 10.1371/journal.pone.0161880

**Published:** 2016-08-25

**Authors:** Haoming Wu, Abinash Padhi, Junqiang Xu, Xiaoyan Gong, Po Tien

**Affiliations:** 1 College of Chemistry and Molecular Sciences, Wuhan University, Wuhan 430072, Hubei, China; 2 College of Life Sciences, Wuhan University, Wuhan 430072, Hubei, China; 3 Department of Animal and Avian Sciences, University of Maryland, College Park, 20742, MD, United States of America; 4 Institute of Microbiology, Chinese Academy of Sciences, Beijing 100101, China; 5 Hubei Provincial Centers for Disease Control and Prevention, Wuhan 430072, Hubei, China; University of Cincinnati College of Medicine, UNITED STATES

## Abstract

The non-pathogenic Human Pegivirus (HPgV, formerly GBV-C/HGV), the most prevalent RNA virus worldwide, is known to be associated with reduced morbidity and mortality in HIV-infected individuals. Although previous studies documented its ubiquity and important role in HIV-infected individuals, little is known about the underlying genetic mechanisms that maintain high genetic diversity of HPgV within the HIV-infected individuals. To assess the within-host genetic diversity of HPgV and forces that maintain such diversity within the co-infected hosts, we performed phylogenetic analyses taking into account 229 HPgV partial E1-E2 clonal sequences representing 15 male and 8 female co-infected HIV patients from Hubei province of central China. Our results revealed the presence of eleven strongly supported clades. While nine clades belonged to genotype 3, two clades belonged to genotype 2. Additionally, four clades that belonged to genotype 3 exhibited inter-clade recombination events. The presence of clonal sequences representing multiple clades within the HIV-infected individual provided the evidence of co-circulation of HPgV strains across the region. Of the 23 patients, six patients (i.e., five males and one female) were detected to have HPgV recombinant sequences. Our results also revealed that while male patients shared the viral strains with other patients, viral strains from the female patients had restricted dispersal. Taken together, the present study revealed that multiple infections with divergent HPgV viral strains may have caused within-host genetic recombination, predominantly in male patients, and therefore, could be the major driver in shaping genetic diversity of HPgV.

## Introduction

Human Pegivirus (HPgV, formerly GBV-C/HGV), a positively single stranded RNA virus of the genus *Pegivirus* (family: *Flaviviridae*) [1], which is the most prevalent non-pathogenic RNA virus worldwide [2], has been reported to be associated with reduced morbidity and mortality in HIV-infected individuals [3–6]. The mechanisms by which HPgV modulated HIV infection include direct interference with HIV entry and replication and indirect regulation of host factors that can ameliorate disease progression [7, 8]. Due to the shared transmission routes, namely through sexual contact [9, 10], blood donation [11] and intravenous drug usage[12], co-infection with HPgV is common among people infected with HIV-1. Relatively high incidence of co-infections with HPgV (≈ 37%) have been reported in the HIV infected subjects in the Hubei province of China [13]. Phylogenetically, HPgV has been classified into five genotypes (genotypes 1–5) [12, 14], and genotype 3 is reported to be predominant in China [13, 15–17]. Despite the presence of HIV, HPgV strains belonging to genotype 3 have been reported to exhibit remarkable population growth within each co-infected host and the E1-E2 genomic regions of HPgV experienced intense purifying selection [13]. Despite these facts, little is known about the forces that contribute to such high genetic diversity of HPgV within the HIV co-infected patients.

Genetic recombination, which is an important evolutionary mechanism in RNA viruses [18–23], is known to be the major driving force in maintaining high genetic diversity in HPgV [24–26]. However, it is unclear to what extent such genetic recombination contributes in maintaining high genetic diversity in HIV infected hosts and whether such viral diversity patterns within each individual are gender-biased. Utilizing the HPgV E1-E2 sequence data from the HIV-HPgV co-infected patients residing in the Hubei province of China, the objective of the present study was to investigate the role of recombination on genetic diversity within each patient, and further to explore whether a patient’s gender has a considerable effect on the HPgV recombination and HPgV viral dispersal across the region.

## Materials and Methods

### Ethics Statement

The patient samples were collected during October 2009 to November 2010, and this research plan was approved by the ethics committees of the Hubei Provincial Institute for Infectious Disease Control and Prevention. All the patients provided written consent, and the documents have been preserved by the ethics committee of Hubei Provincial Institute for Infectious Disease Control and Prevention.

### Sample Collection, RNA Extraction, PCR and Sequencing

HIV positive samples analyzed in this study were tested for the presence of HPgV RNA using primers from the 5′-UTR [13]. Twenty-three previously identified HIV/HPgV co-infection patients from 13 counties of Hubei province in China were enrolled in this study. Total RNA was extracted from 100 μl serum with Trizol LS reagents (Invitrogen, Carlsbad, California, USA) following the manufacturer’s instructions. Reverse transcription was carried out with random hexamers primers (Promega, Madison, Wisconsin, USA), M-MLV reverse transcriptase (Promega, Madison, Wisconsin, USA), ribonuclease inhibitor (Biostar International, Canada) and 2 μg eluted RNA in a total volume of 25 μl for 60 min at 37°C, following a preheating step for 10 min at 70°C. The 906bp length sequences of HPgV covering partial E1 region and E2 region (positions 963–1868, corresponding to the GenBank accession: AF121950) was amplified using high fidelity DNA Polymerase Pyrobest (Takara, Japan). The amplification of E1-E2 region was performed by nested PCR using outer primers (E2_F: 5′-RGTGGGRRAGTGAGTTTTGGAGAT-3′ and E2_R1: 5′-RAACGTHCCRGTVGGAGGCT-3′) and inner primers (E1fon: 5′-TGGGAAAGTGAGTTTTGGAGATGG-3′ and E2_R2: 5′-DTCYCGGATCTTGGTCATGG-3′). The touchdown PCR reaction was initiated with a preheating procedure (95°C for 5 min) and performed for 30 cycles (the annealing temperature was progressively lowered from 65°C to 50°C by 1°C every cycle, followed by 15 additional cycles at 50°C) and a final extension cycle at 72°C for 10 min on a thermocycler. Subsequently, PCR products were extracted from the gel using the Easy Pure Quick Gel Extraction Kit (TransGen Biotech, Beijing, China) and then were TA-cloned into plasmid pTA2 vector using the Target CloneTM kit (Toyobo, Osaka, Japan) following the manufacturer’s instructions. After an incubation period of 24 h on LB agar plates in the presence of 50 μg/ml ampicillin, the resultant clones were screened for the proper insert based on the color reaction using the Xgal-IPTG system. Nine to ten clones of each patient were randomly picked up for sequencing. Sequencing was carried out using the ABI-PRISM3730 sequencer in Sangon Biotechnology of China. All the sequences generated in this study were deposited in GenBank (accession numbers KU843606-KU843834). To evaluate the nucleotide variability originated from PCR error, a known sequence was PCR amplified, cloned, and sequenced under identical conditions. Ten independent clones were analyzed and showed absolute identity with the parental sequence.

### Recombination Analysis

Coding nucleotide sequences of the E1-E2 genomic region were aligned using the MUSCLE algorithm implemented in MEGA 7 [27]. Using the same program nucleotide diversity for each patient and the corresponding standard errors were also estimated. To detect the potential recombinant sequences in the dataset, we performed three independent analyses using the methods implemented in SplitsTree ver. 4 [28], RDP4 [29], and Simplot v3.5.1 programs [30]. SplitsTree ver. 4 is based on the well-established pairwise homoplasy test in conjunction with split-decomposition networks and was used to identify the presence of recombination in our dataset. Split networks were generated with the Neighbor Net algorithm and the pairwise homoplasy indexes (PHI) of the networks were calculated. An observed PHI value < 0.05 indicated significant presence of recombination. The split networks generalize phylogenetic trees that allow the representation of conflicting signals or alternative evolutionary history, including recombination events. Putative recombinants are usually located at parallel edges in the network. By progressively removing sequences at the vertices, we would find which sequence could significantly increase the *p*-value by PHI test. Until the *p*-value was > 0.05, sequences that are removed could be considered as putative recombinants.

Detection of potential recombinant sequences and putative breakpoint events were also carried out using the RDP, GENECONV, Maxchi, Chimaera, Siscan, and 3Seq algorithms implemented in RDP4 software package. Sequences are considered to be recombinant if the *p*-values < 0.05 after Bonferroni correction for multiple tests. The breakpoint positions and recombinant sequences inferred for each detected potential recombination event were manually checked. The SimPlot v3.5.1 program was also used to identify the putative recombination breakpoints. The recombinants were confirmed using a boot-scanning analysis with 1000 bootstrap replicates. The window size and the step size were set to 200bp and 20bp, respectively. Statistical tests such as Fisher’s exact test for categorical variables and Welch’s two sample t-test for continuous variables were performed using R ver. 3.10 [31].

### Phylogenetic Analysis

The Maximum-likelihood (ML) tree was reconstructed under the appropriate nucleotide substitution model using the MEGA7 [27]. Using the same program, robustness of the tree was evaluated by the bootstrapping with 1000 replicates. To determine the genotype affiliation of each viral clone, reference sequences were retrieved from GenBank and were included in the phylogenetic analysis. A chimpanzee HPgV variant (SPgV_tro_) (GenBank accession: AF070476) was used as the outgroup. The best-fit model of nucleotide substitution was selected according to the Bayesian Information Criterion (BIC) implemented in MEGA7 [27]. The General Time Reversible (GTR) with invariable sites (I) and gamma distribution (Γ) parameter is the best-fit model. Additionally, to assess the robustness of ML tree topologies, we also estimated posterior probabilities for each node by performing BMCMC (Bayesian Markov Chain Monte Carlo) analyses implemented in MrBayes, version 3.1.2 [32]

## Results

### HPgV Infection Status

In the present study, a total of 229 clonal sequences, each with a nucleotide sequence length of 906-bp covering partial E1-E2 gene of HPgV, from 23 HIV/ HPgV co-infected patients (15 males; 8 females) with the ages ranging from 24 to 58 years residing in the Hubei province of central China were analyzed. The number of clones, infection status, and viral transmission routes of each patient are listed in Table 1. The 23 patients were tested as positive for HPgV RNA and negative for anti-E2 antibody. In addition, none of the patients were infected with HBV and HCV.

**Table 1:** Number of patients, patient ID, transmission route, CD4 count, HIV viral load, number of clones, and Phi test for each patient analyzed in this study.

Patient ID^a^	Infection Route	Therapy	CD4	Viral Load^b^	Viral sequences		PHI Test
					Total	Recombinants	
JYM_1	heterosexual	No	670	<LDL	10	0	0.07865
CBM_2	heterosexual	HAART	221	<LDL	10	0	NI^c^
QCF_3	paid blood donor	HAART	405	<LDL	10	0	0.3697
JZM_7	paid blood donor	No	429	UN	10	0	1.0
CBM_8	homosexual	No	594	UN	10	0	0.03394
QCM_9	heterosexual	No	16	13900	10	0	1.0
XAM_10	heterosexual	HAART	255	<LDL	10	0	1.0
JZM_23	heterosexual	No	695	UN	10	0	1.0
**XAM_27**	**homosexual**	**No**	**36**	**3030**	**10**	**4**	**0**
**QCM_31**	**paid blood donor**	**HAART**	**261**	**<LDL**	**10**	**7**	**0**
**QCM_32**	**heterosexual**	**HAART**	**259**	**<LDL**	**10**	**1**	**0.581**
CBF_33	intravenous drug	No	49	178	10	0	1.0
QCF_34	paid blood donor	HAART	411	<LDL	10	0	NI
QCF_35	heterosexual	HAART	193	<LDL	10	0	0.1103
CYM_36	heterosexual	No	369	UN	9	0	1.0
**TSF_37**	**heterosexual**	**HAART**	**188**	**<LDL**	**10**	**5**	**0**
CYF_38	heterosexual	HAART	366	<LDL	10	0	1.0
**JZM_39**	**homosexual**	**HAART**	**373**	**<LDL**	**10**	**4**	**0**
**CYM_40**	**heterosexual**	**No**	**238**	**UN**	**10**	**1**	**NI**
CYF_41	heterosexual	HAART	318	<LDL	10	0	1.0
JZM_46	homosexual	HAART	345	<LDL	10	0	NI
TCF_55	heterosexual	HAART	296	<LDL	10	0	0.4319
JZM_58	heterosexual	HAART	407	<LDL	10	0	0.007965

^a^The nomenclature of patients. The first two letters represent sampling locations; the last letter represents the gender of each patient: M for male and F for female; the numeric represent patients' code.

^b^HIV viral load (RNA copies/ml); UN: undection; <LDL: below the lower detection limit.

^c^There are too few informative characters to use the Phi Test as implemented here.

Patients with recombinant sequences are in bold.

### Within-Host Genetic Recombination and Phylogenetic Analysis

**Fig 1:**
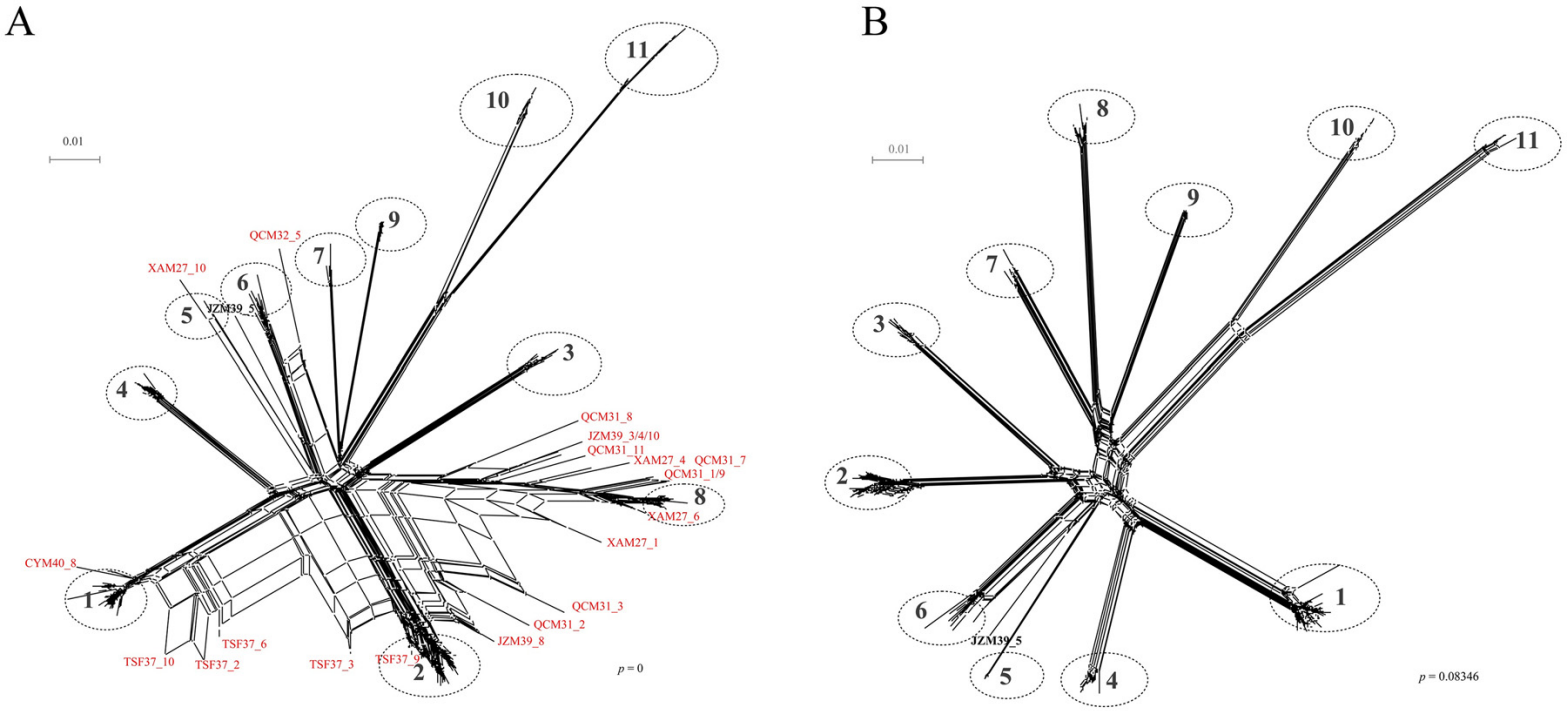
Phylogenetic networks of HPgV E1-E2 segments. (**A**) A split network, including all sequences, was first constructed. The sequences clustered in eleven clades designated 1 to 11. The outliers are classified as putative intra-subtype recombinants (in red) or a novel clade (in black). (**B**) Sequences inferring conflicting phylogenetic signals were excluded (recombinant candidates) were removed, and a new split network was constructed.

**Fig 2:**
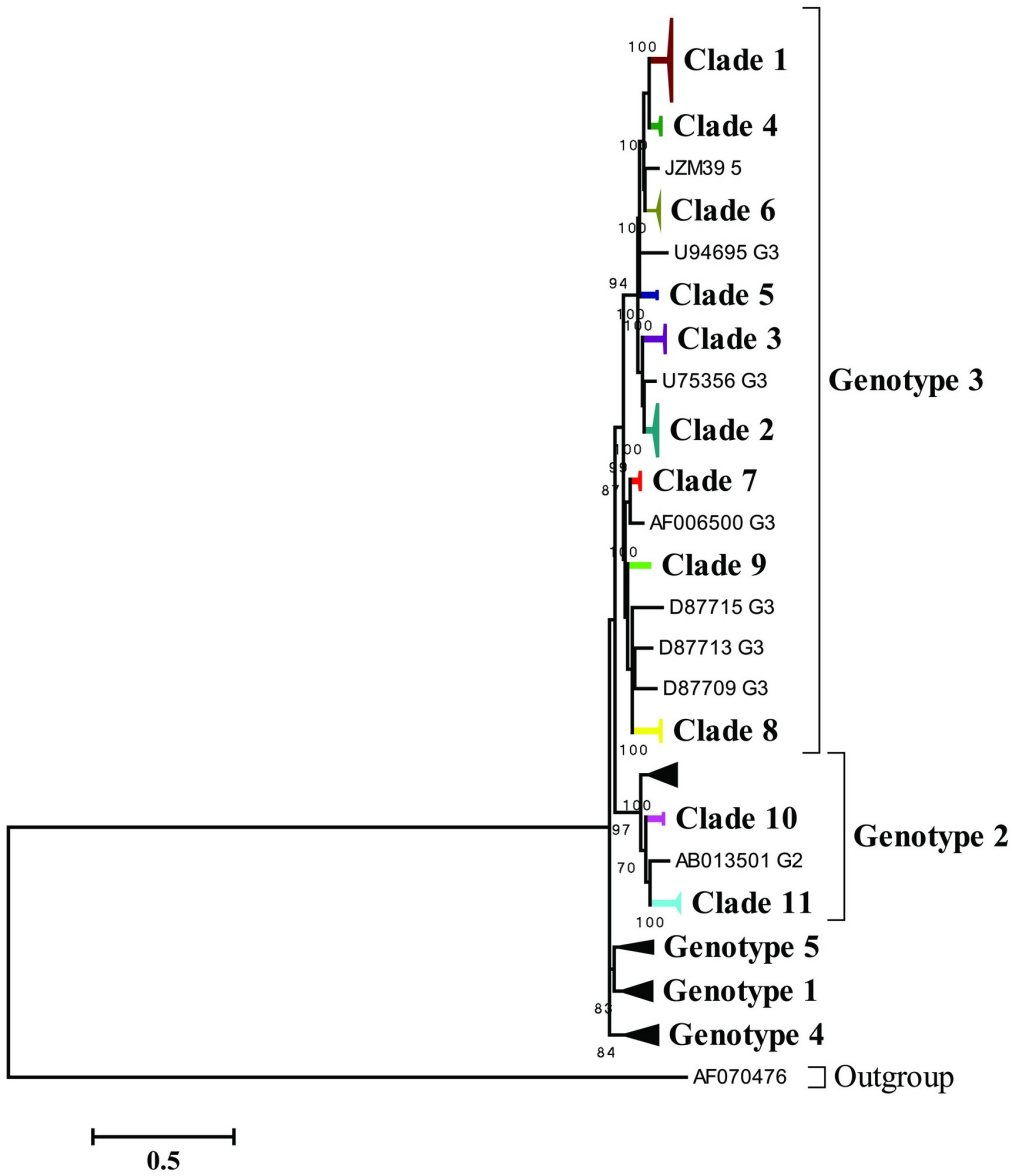
Maximum likelihood tree depicting two and nine clades belong to genotype 2 and genotype 3, respectively. Nodes with bootstrap values > 70 are mentioned at the base of the nodes. Recombinant sequences were excluded from the analyses. Clades that were recovered in the present study are shown in different colors. GenBank reference sequences representing genotypes 1 to 5 are included in the analyses.

A split network was initially constructed using all the 229 sequences (Fig 1A). These sequences fall into eleven major divergent clades (clades: 1–11) and exhibited a reticulate topology (Fig 1A). Some sequences from patients XAM_27, QCM_31, JZM_39, TSF_37, CYM_40 and QCM_32 emerged as outliers and formed branches of the major clades (Fig 1A), thus indicating the presence of recombinant sequences. After exclusion of these potential recombinant sequences (i.e., the branches), which yielded conflicting phylogenetic signals, a new split network was constructed to illustrate the evolutionary relationships among the clades (Fig 1B). Consistently, the new network also revealed the same number of clades (clades: 1–11, Fig 1B), however, with no statistical significance for recombination (*p* > 0.05). In contrast to the reticulate topology (Fig 1A), the new network showed a star-like topology (Fig 1B). Consistently, the ML (Fig 2) and the Bayesian (S1 Fig) phylogenies also revealed the presence of 11 clades. Our analyses revealed that while nine clades that comprised of clonal sequences, mostly from 21 patients, belonged to HPgV genotype 3, two clades that comprised of clonal sequences from two patients belonged to genotype 2 (Fig 2). The sequence JZM39_5 formed a novel clade (Figs 1 and 2).

To further define the putative recombination signals as observed by PHI test, we also analyzed the data using the distance-based methods implemented in recombination detection program RDP4 (Table 2) and SimPlot (S2 Fig). Consistent with the split network results (Fig 1A), RDP4 also showed strong evidence of recombination events and were detected in the same six patients (i.e., patients XAM_27, QCM_31, JZM_39, TSF_37, CYM_40 and QCM_32). Interestingly, five of the six patients were male. Based on these results, it appears that genetic recombination may occur more frequently in male patients than the female patients; however, due to limited sample size, this result should be interpreted with caution. Most of the deduced parental recombinant sequences belonged to clades 1, 2 and 8 (Table 2). With the exception of few clades (clades: 1, 2 and 8), most of the clades are represented by the clonal sequences of female patients with no evidence of sharing of the sequences among these female patients (Table 3). In contrast, while clade 1 and 8 shared sequences among the male patients, clade 2 shared the sequences between male and female patients (Table 3). Altogether, these results clearly indicate co-circulation of phylogenetically distinct strains, and therefore, indicating the possibility of inter-clade recombination within each patient.

**Table 2:** Summary of unique recombination events identified by RDP4 program.

Patient ID	Recombination Event Number	Breakpoint Positions	Recombinant Sequences	Minor Parental Sequence(s)	Major Parental Sequence(s)^a^	Detection Methods					
						RDP	GENECONV	Maxchi	Chimaera	SiSscan	3Seq
XAM_27	1	468–720	XAM27_4	XAM27_2/3/5/9(clade 8)	XAM27_7/8(clade 5)	-	9.39E-10	7.52E-10	4.46E-10	8.22E-10	7.74E-21
	2	242–742	XAM27_1	XAM27_7/8(clade 5)	XAM27_2/3/5/9(clade 8)	-	3.00E-04	2.33E-09	2.33E-09	1.03E-12	1.53E-16
	3	342–516	XAM27_10	XAM27_7/8(clade 5)	XAM27_2/3/5/9(clade 8)	-	1.25E-02	2.11E-02	-	-	1.32E-07
	4	742–884	XAM27_6	XAM27_2/3/5/9(clade 8)	XAM27_7/8(clade 5)	-	9.20E-04	2.67E-02	-	-	5.72E-05
JZM_39	1	592–701	JZM39_3	JZM39_5	JZM39_1/2/6/7/9(clade 8)	6.24E-05	1.15E-05	5.84E-09	1.94E-08	1.30E-09	4.82E-10
	2	358–742	JZM39_3/10	JZM39_8(clade 2)	JZM39_1/2/6/7/9(clade 8)	-	6.81E-05	1.73E-10	7.97E-09	8.01E-14	1.68E-16
	3	376–814*	JZM39_4	JZM39_5	JZM39_1/2/6/7/9(clade 8)	-	2.79E-05	1.62E-05	1.53E-05	8.29E-07	8.63E-11
	4	1–178	JZM39_8	JZM39_1/2/6/7/9(clade 8)	clade 2	2.60E-06	-	5.04E-07	4.34E-04	1.45E-19	8.76E-12
QCM_31	1	430–745	QCM31_3	QCM31_4/10(clade 8)	QCM31_6(clade 2)	1.68E-07	2.41E-06	9.01E-10	1.28E-10	6.42E-10	1.19E-20
	2	516–662	QCM31_7	QCM31_6(clade 2)	QCM31_4/10(clade 8)	8.84E-12	4.44E-10	5.88E-07	4.84E-07	5.17E-09	1.76E-15
	3	70–309	QCM31_2/8	QCM31_4/10(clade 8)	QCM31_6(clade 2)	2.78E-03	8.09E-06	8.97E-10	5.65E-07	-	1.57E-14
	4	69*-682	QCM31_1/9	QCM31_6(clade 2)	QCM31_4/10(clade 8)	-	7.78E-05	4.00E-04	1.11E-03	-	3.12E-11
	5	544–780	QCM31_11	QCM31_6(clade 2)	QCM31_4/10(clade 8)	7.78E-07	5.83E-06	1.37E-09	1.75E-07	8.31E-03	5.14E-14
	6	508–701	QCM31_8	JZM39_5	QCM31_6(clade 2)	-	2.62E-03	1.41E-05	1.30E-05	7.82E-07	1.36E-08
TSF_37	1	72*-187	TSF37_2	TSF37_1/4/5/8(clade 2)	TSF37_7(clade 1)	-	8.89E-05	-	-	1.98E-13	2.27E-07
	2	574–853*	TSF37_3	TSF37_7(clade 1)	TSF37_1/4/5/8(clade 2)	-	5.47E-05	7.70E-08	1.52E-07	5.41E-08	5.02E-14
	3	3–259*	TSF37_6	TSF37_1/4/5/8(clade 2)	TSF37_7(clade 1)	-	1.72E-02	-	-	-	4.36E-08
	4	770–865	TSF37_9	TSF37_7 (clade 1)	TSF37_4(clade 2)	-	2.40E-02	1.91E-04	1.50E-02	-	3.97E-10
	5	648–870	TSF37_2/10	TSF37_1/4/5/8(clade 2)	TSF37_7(clade 1)	-	3.57E-08	1.47E-04	1.47E-04	-	1.12E-12
CYM_40	1	502–682*	CYM40_8	clade 2	CYM40_X(clade 1)	-	1.49E-05	1.80E-05	1.80E-05	-	1.08E-11
QCM_32	1	180–363	QCM32_5	Unknown	QCM32_X(clade 6)	-	-	3.27E-02	-	6.60E-13	1.29E-02

* = The actual breakpoint position is undetermined (it was most likely overprinted by a subsequent recombination event).

- = No significant P-value was recorded for this recombination event using this method.

^a^ X = The remaining non-recombination sequences of the patient.

**Table 3:** The number of sequences shared by the patients for a given clade after the exclusion of recombinant sequences.

Patient ID	Genotype 3									Genotype 2	
	1	2	3	4	5	6	7	8	9	10	11
JYM_1	8							1			
CBM_2	10										
JZM_7	10										
CBM_8	10										
QCM_9	8		2								
XAM_10	9	1									
**CYM_40**	9										
CBF_33		10									
QCF_34		10									
QCF_35		10									
**TSF_37**	1	4									
TCF_55			10								
CYF_38				10							
JZM_58	2			8							
**XZM_27**					5			2			
**QCM_32**						9					
CYF_41							10				
**JZM_39**								5			
JZM_46	1							9			
**QCM_31**		1						2			
CYM_36		2							7		
JZM_23										10	
QCF_3											10

Patients with recombinant sequences are in bold.

**Fig 3:**
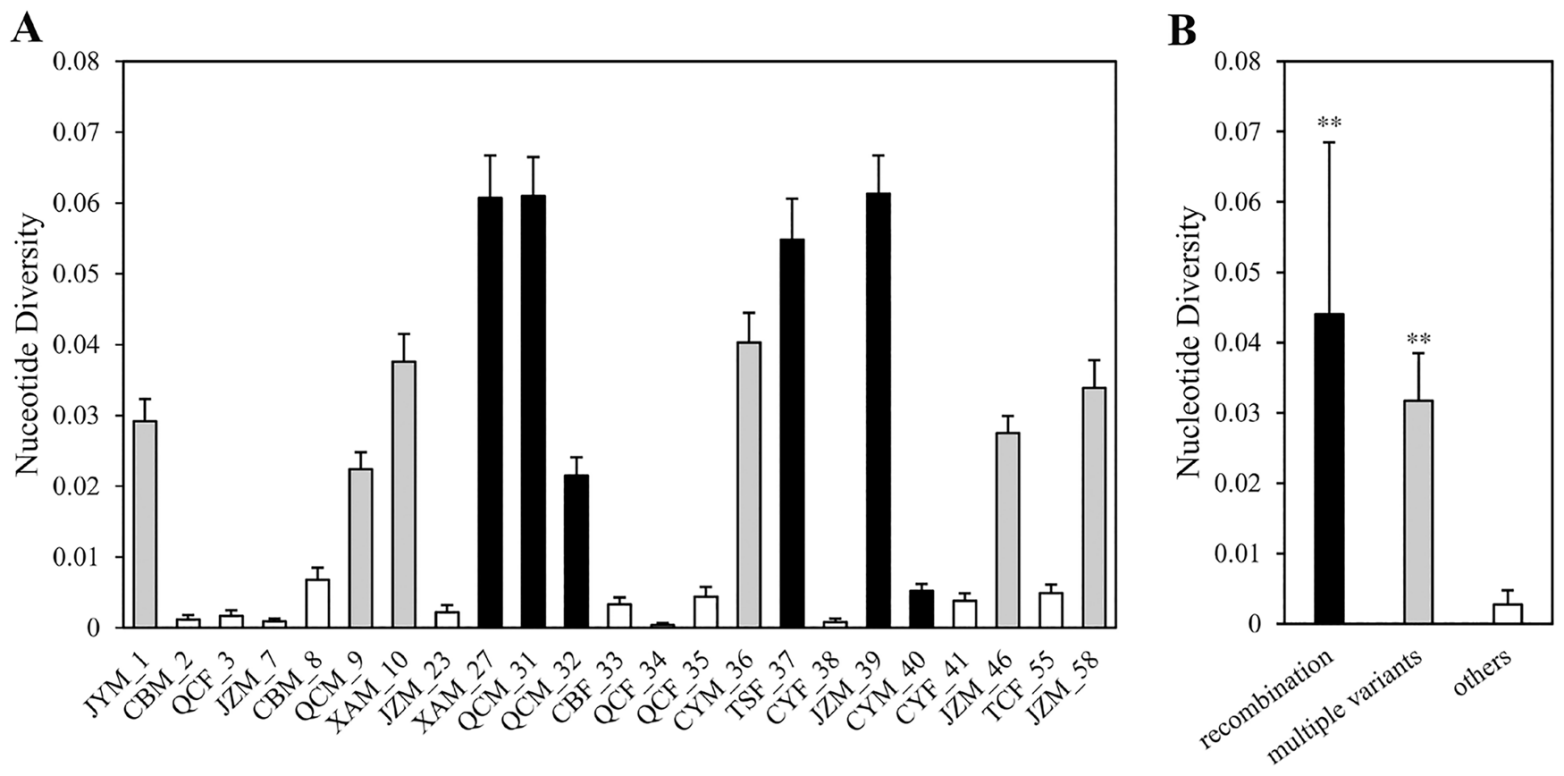
Nucleotide diversity of each patient. The bar plots for each individual (**A**) and patient-specific group (**B**) show a relatively higher intra-host nucleotide diversity in patients detected with recombination sequences (black) or/and infected with multiple variants (grey) than other patients (white). Nucleotide diversity was evaluated regardless of recombination.

If all the viral clones within each patient have a single origin, these clones are expected to exhibit patient-specific clustering [13]. Therefore, the observation of such patient-specific clustering was also expected in some patients (Fig 1 and Table 3). However, sequences from ten patients (9 males *vs* 1 female) still appeared to be non-monophyletic and are shared among the patients, including the four recombinant patients XAM_27, QCM_31, JZM_39 and TSF_37. Interestingly, the nucleotide diversity of HPgV in each of these four patients is relatively higher than the other patients (black bar in Fig 3; *p* <0.01), implying that recombination played an important role in shaping HPgV diversity. Additionally, the other six patients who were infected with viral strains representing multiple clades of HPgV (Table 3) also have a relative higher nucleotide diversity (grey bar in Fig 3; *p* < 0.0001), which indicates the co-circulation of viral strains representing multiple clades.

## Discussion

Genetic recombination, more importantly in (+) ss RNA viruses, played a dominant role in the emergence of new viral strains with new genetic makeup and greater fitness [33]. Knowledge of the recombination mechanisms, host-factors, as well as their putative roles in the emergence divergent HPgV strains, especially in the HIV co-infected individuals, would provide important insights into the understanding of within-host viral evolution. In this study, we report the evidence of intra-genotypic recombination events that are likely to play the dominant role in generating high within-host HPgV genetic diversity. Although HPgV E1-E2 glycoprotein genomic region is constrained by purifying selection [13] and also the viral genome is characterized by the presence of abundant RNA secondary structure motifs [34, 35], recombination can potentially acquire genetic diversity by generating combinations of preexisting nucleotide polymorphisms, and recombination could also act to shuffle the partial or entire RNA secondary structure region to accelerate the process. Consistent with the results of the present study, previous studies have reported that genetic recombination is the likely cause for the observation of the phylogenetic incongruence among the subgenomic regions of HPgV [36, 37], thus suggesting that genetic recombination has a profound influence on the genomic architecture of HPgV.

Further, our results also provide the evidence of within-host (predominantly in male patients) inter-clade recombination between the divergent strains of HPgV belonging to genotype 3. The presence of divergent strains of HPgV indicates that these patients might have been infected with the HPgV strains multiple times. Given the same transmission routes as HIV, having multiple sexual partners with unprotected sex may increase the chance of being infected with multiple yet divergent strains of HPgV [38]. It may be possible that individual patients might have acquired HPgV strains multiple times through sexual contacts even prior to infection with HIV. Taken together, multiple infections with divergent HPgV viral strains and within-host genetic recombination, could be the major drivers in shaping genetic diversity of HPgV, and also might have contributed to the remarkable population growth of HPgV strains within each HIV/HPgV co-infected host [13].

Males and females inherently differed in their susceptibility, multiplicity infection and virus diversity to a variety of DNA and RNA viruses [39–41]. Previous studies reported that biological and genetic differences in immune responses and exposure to viruses may contribute to the gender-bias susceptibility [42, 43]. In this study, five of the six patients who had recombinant sequences were male. However, due to limited sample size, we could not draw a conclusion as to whether a patient’s gender played an important role in HPgV recombination. Interestingly, previous studies have reported the male-dominant prevalence of HPgV [44]. High risk behaviors, such as male-to-male sex (MSM), are also likely to be associated with a high prevalence of persistent HPgV infection [9, 45]. Although the data may greatly vary according to geographic regions and social culture, males seem to have higher incidence of high-risk sexual exposure than females [46, 47]. To some extent, this pattern is also consistent with the available statistical data, for instance, statistics from the National Survey of Family Growth (NSFG) (available at: http://www.cdc.gov/nchs/nsfg/key_statistics/n.htm).

Collectively, our study provides evidence of within-host genetic recombination in HPgV in HIV co-infected individuals, and also suggests that infection with multiple variants dramatically increases the overall viral diversity. While the biological consequences of viral diversity and recombination of HPgV have not been examined, studies of HIV and HCV suggest that viral diversity and recombination may result in altered cell tropism, virulence, immune evasion and drug resistance/sensitivity [48–51]. Nevertheless, the present study indicates that the observation of male-dominant recombination patterns and high HPgV viral diversities may likely be associated with the higher incidence of high-risk sexual exposure of the male patients. However, due to the limited sample size, the inference of the male-dominant recombination patterns should be taken with caution. Further study with larger cohorts of samples is required to validate the male-dominant recombination patterns as observed in the present study.

## Supporting Information

S1 Fig Bayesian phylogeny depicting two and nine clades belonged to genotype 2 and genotype 3, respectively. Nodes with posterior probabilities > 0.9 are mentioned at the base of the nodes. Recombinant sequences were excluded from the analyses. Clades that were recovered in the present study are shown in different colors. GenBank reference sequences representing genotypes 1 to 5 are included in the analyses.
(TIF)

S2 Fig SimPlot analysis for the HPgV E1-E2 segments. The bootscanning plot was carried out on each putative recombinant. The parameters used for analysis are shown on the bottom row of each figure. The QCM32_5 was not included in the bootscanning analysis for the unidentified minor parental sequences.
(ZIP)
