# Supplementary figures and images for: Evidence for Within-Host Genetic Recombination among the Human Pegiviral Strains in HIV Infected Subjects
Source: PLoS One. 2016 Aug 25;11(8):e0161880. doi: 10.1371/journal.pone.0161880 (PMC4999292; doi:10.1371/journal.pone.0161880)

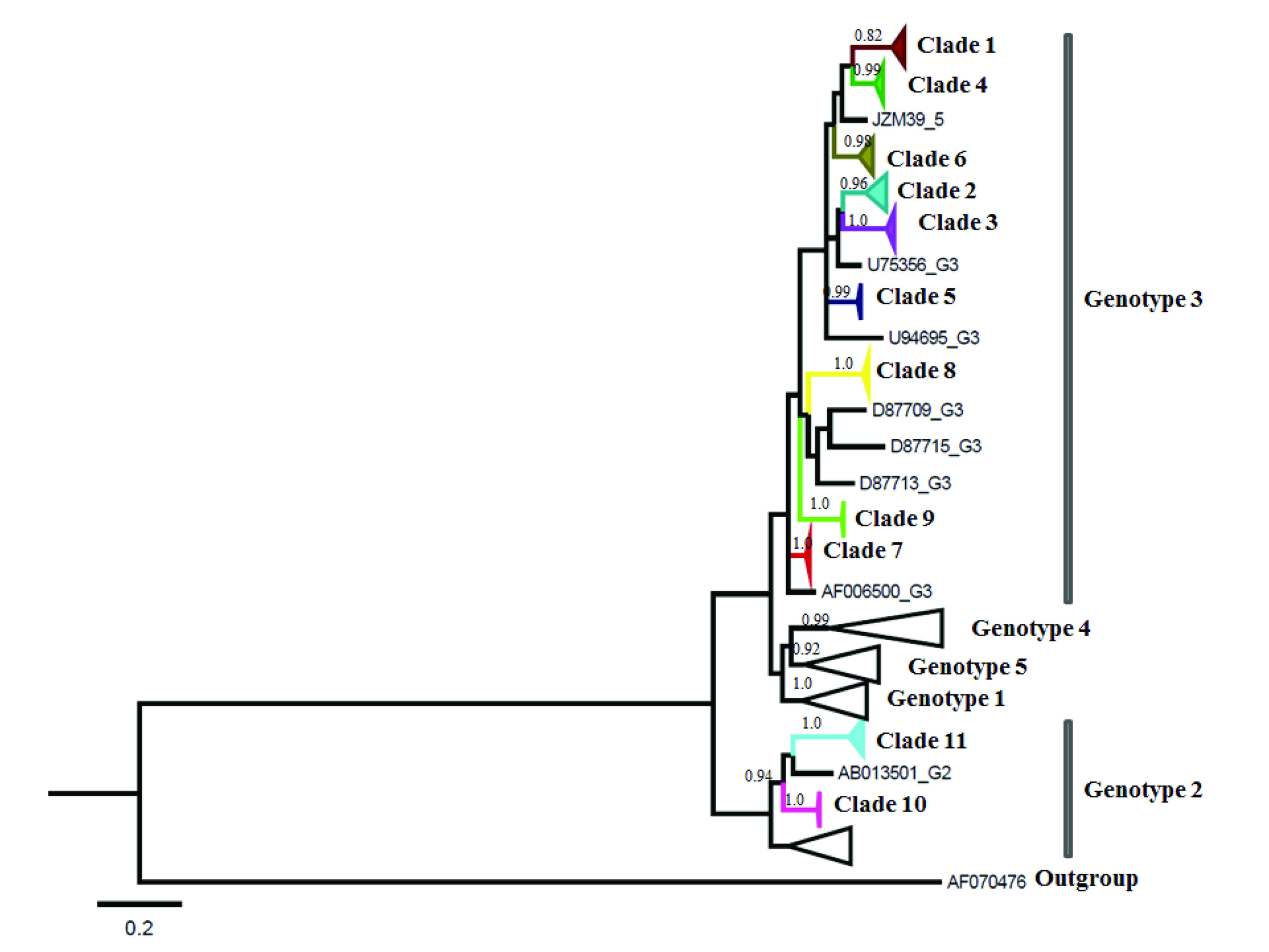

Supplement: S1 Fig — Nodes with posterior probabilities > 0.9 are mentioned at the base of the nodes. Recombinant sequences were excluded from the analyses. Clades that were recovered in the present study are shown in different colors. GenBank reference sequences representing genotypes 1 to 5 are included in the analyses. (TIF) [file pone.0161880.s001.tif]

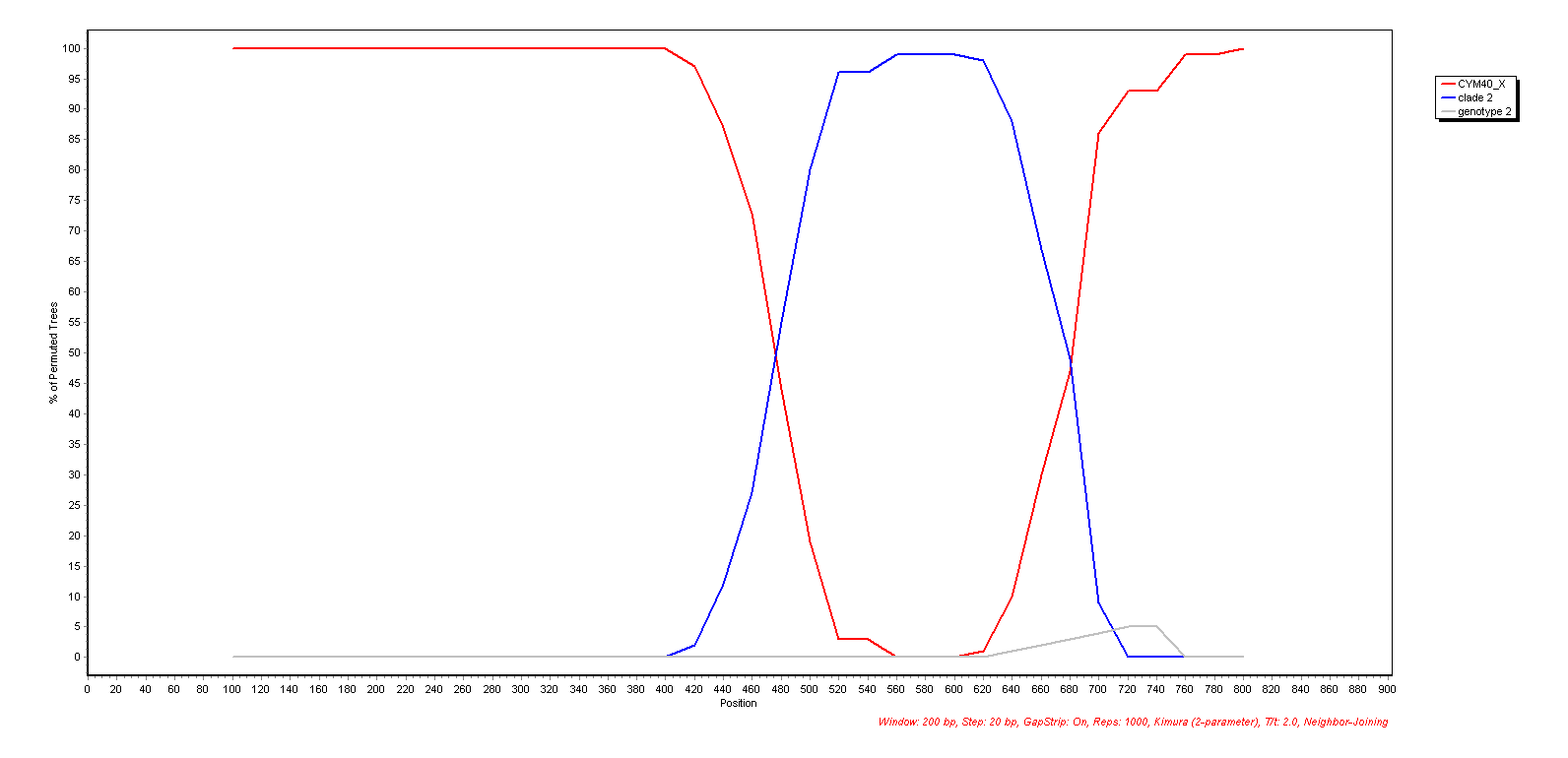

Supplement: S2 Fig — The bootscanning plot was carried out on each putative recombinant. The parameters used for analysis are shown on the bottom row of each figure. The QCM32_5 was not included in the bootscanning analysis for the unidentified minor parental sequences. (ZIP) [file pone.0161880.s002.zip › Supplemental Fig S2/CYM40_8.tif]

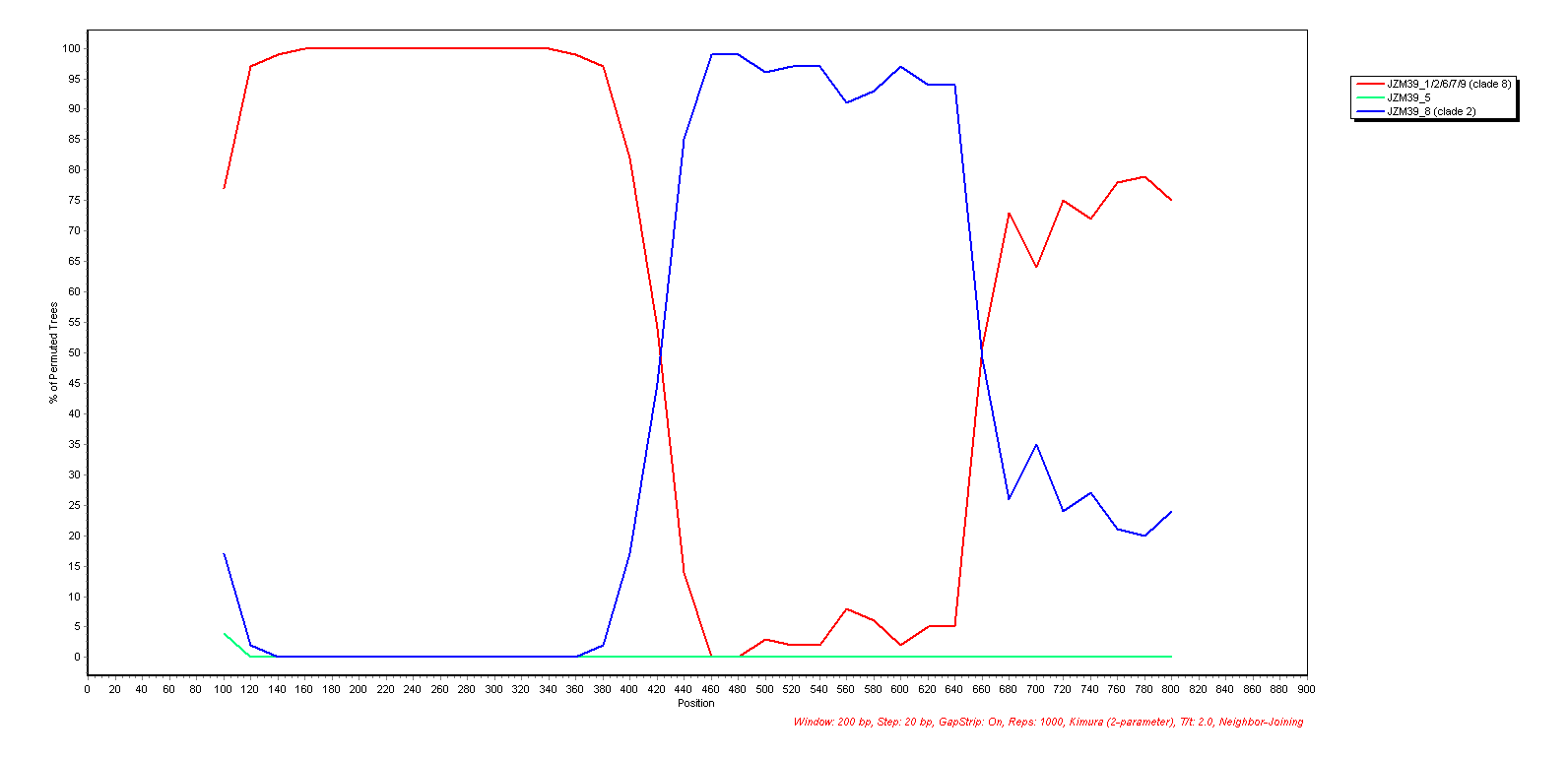

Supplement: S2 Fig — The bootscanning plot was carried out on each putative recombinant. The parameters used for analysis are shown on the bottom row of each figure. The QCM32_5 was not included in the bootscanning analysis for the unidentified minor parental sequences. (ZIP) [file pone.0161880.s002.zip › Supplemental Fig S2/JZM39_10.tif]

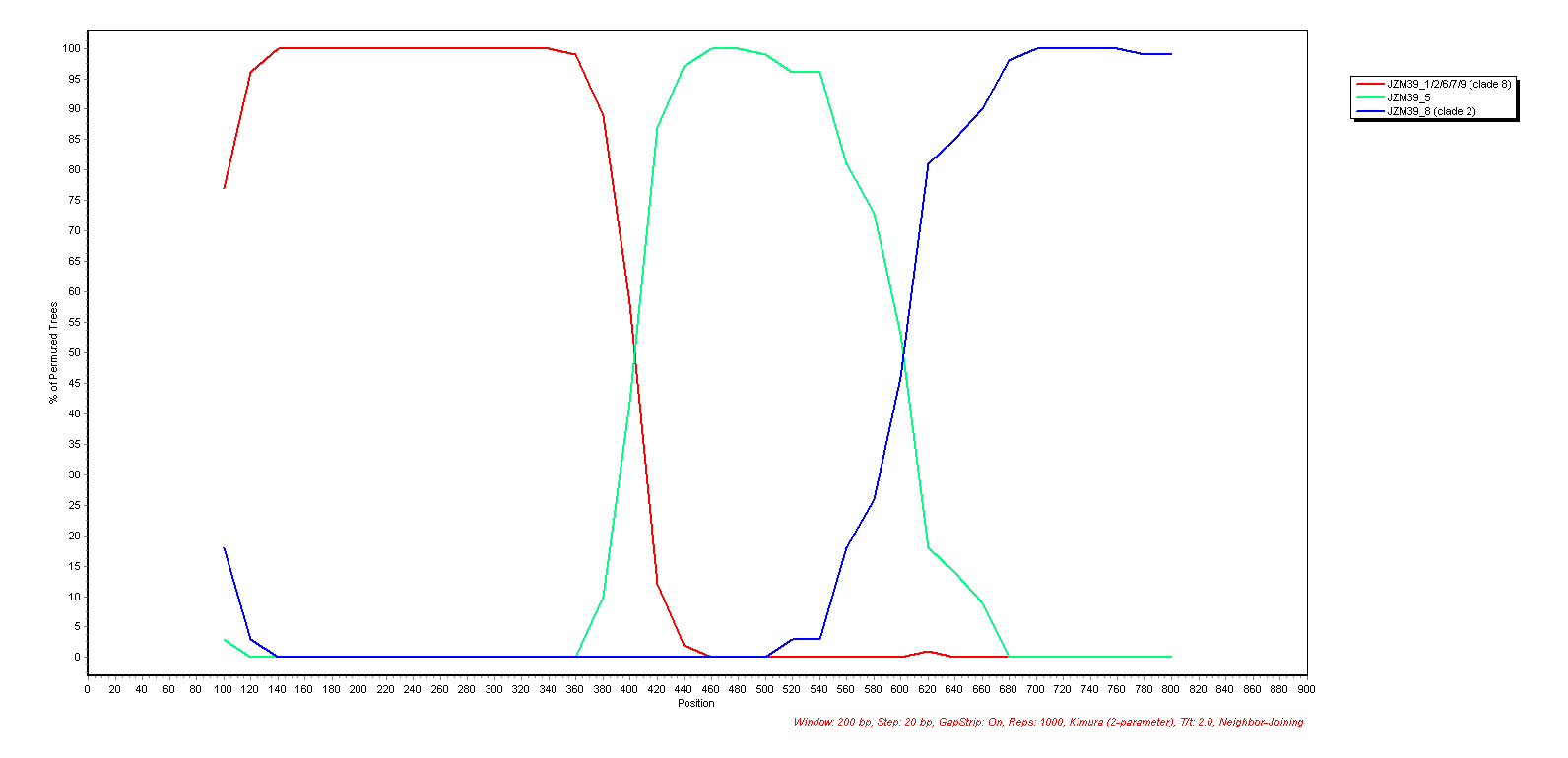

Supplement: S2 Fig — The bootscanning plot was carried out on each putative recombinant. The parameters used for analysis are shown on the bottom row of each figure. The QCM32_5 was not included in the bootscanning analysis for the unidentified minor parental sequences. (ZIP) [file pone.0161880.s002.zip › Supplemental Fig S2/JZM39_3.tif]

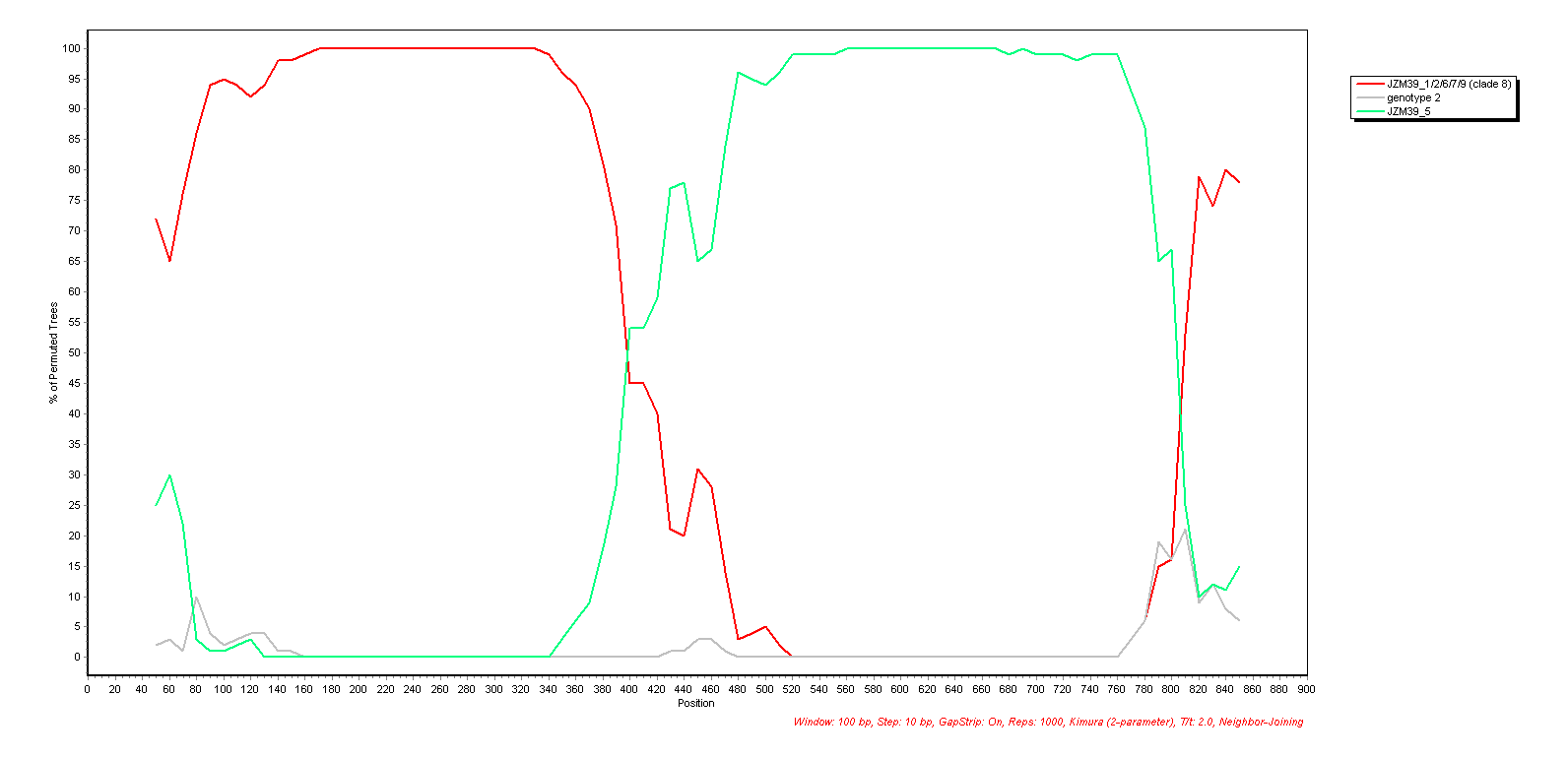

Supplement: S2 Fig — The bootscanning plot was carried out on each putative recombinant. The parameters used for analysis are shown on the bottom row of each figure. The QCM32_5 was not included in the bootscanning analysis for the unidentified minor parental sequences. (ZIP) [file pone.0161880.s002.zip › Supplemental Fig S2/JZM39_4.tif]

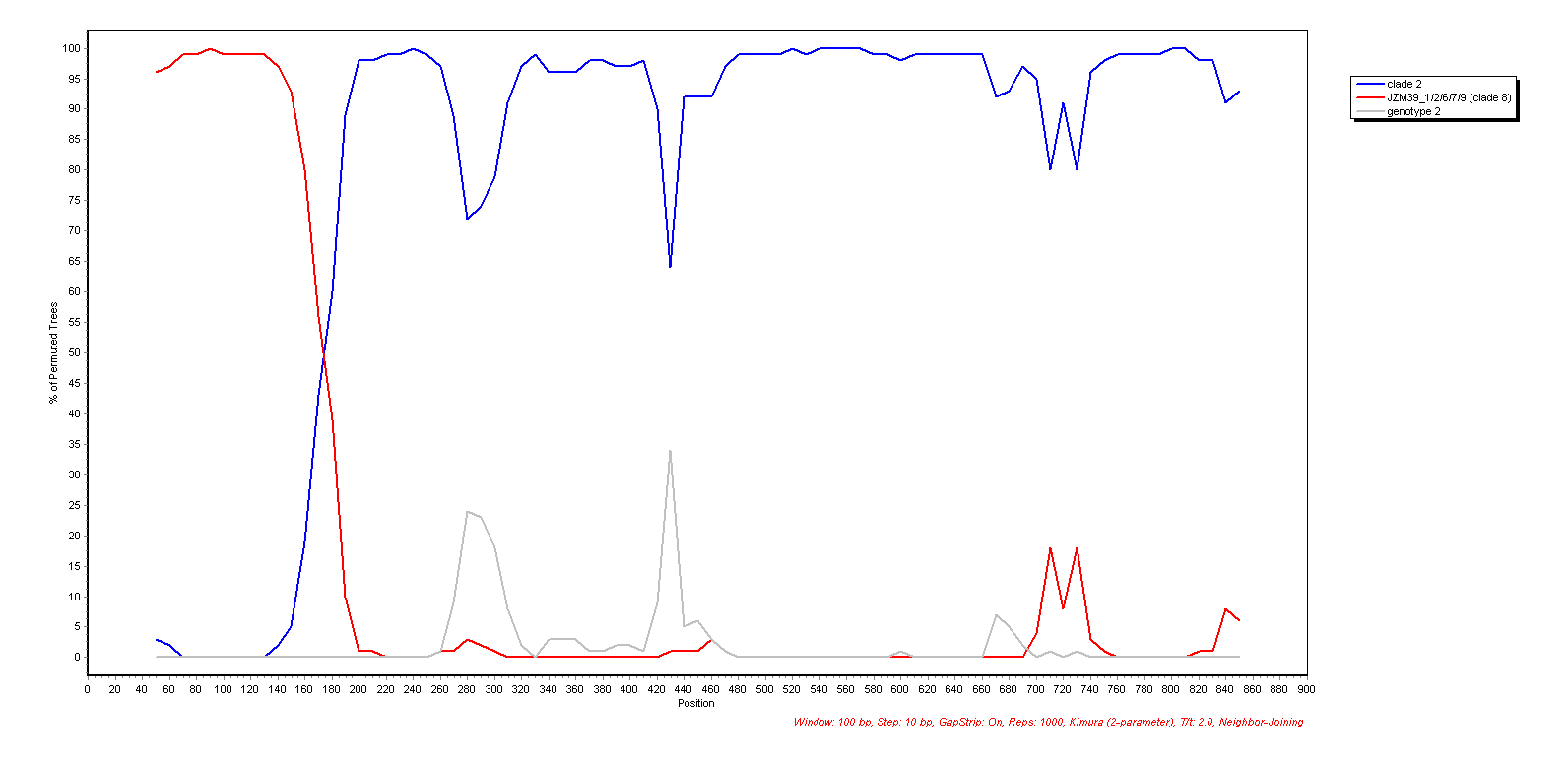

Supplement: S2 Fig — The bootscanning plot was carried out on each putative recombinant. The parameters used for analysis are shown on the bottom row of each figure. The QCM32_5 was not included in the bootscanning analysis for the unidentified minor parental sequences. (ZIP) [file pone.0161880.s002.zip › Supplemental Fig S2/JZM39_8.tif]

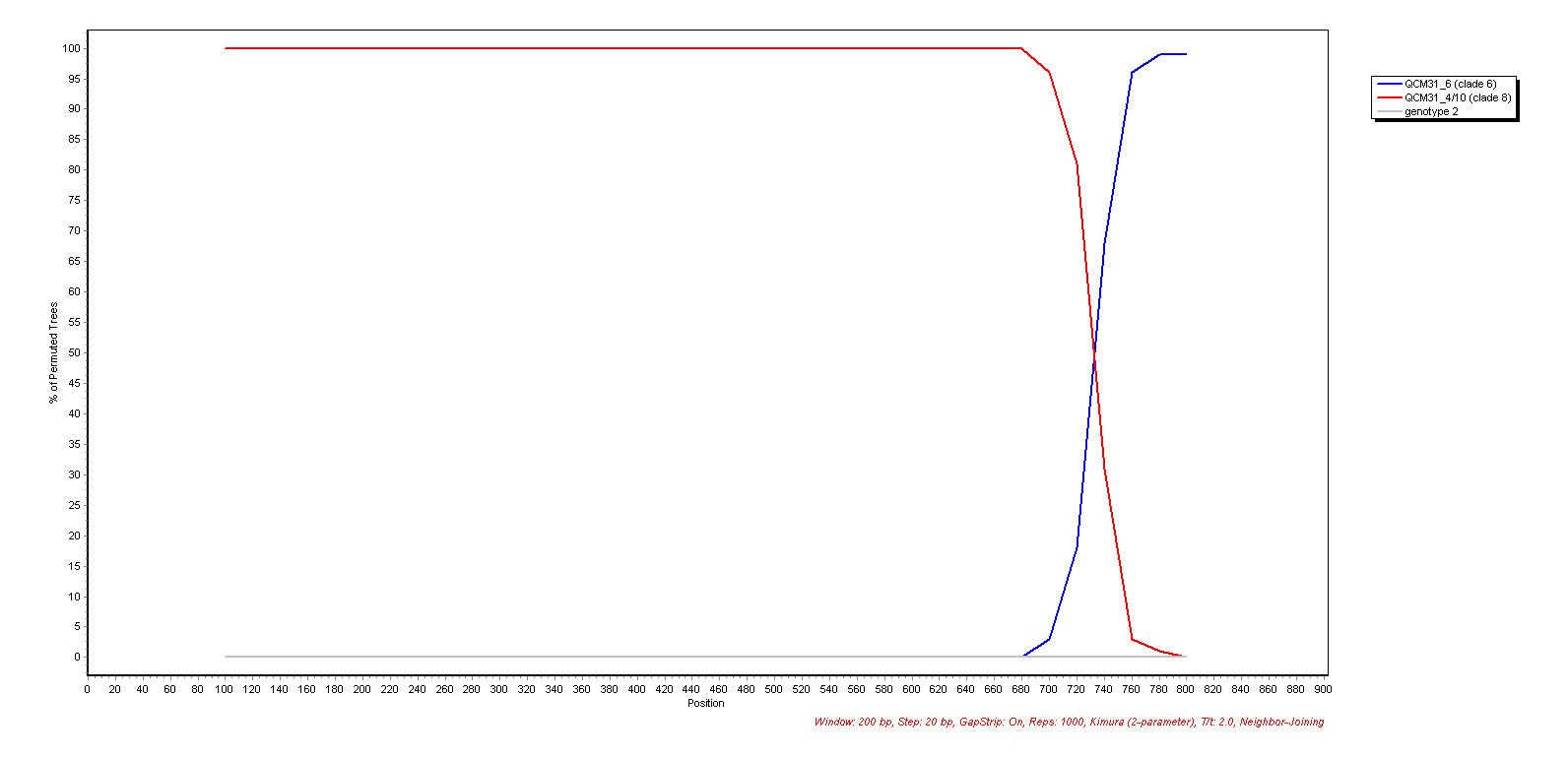

Supplement: S2 Fig — The bootscanning plot was carried out on each putative recombinant. The parameters used for analysis are shown on the bottom row of each figure. The QCM32_5 was not included in the bootscanning analysis for the unidentified minor parental sequences. (ZIP) [file pone.0161880.s002.zip › Supplemental Fig S2/QCM31_1+9.tif]

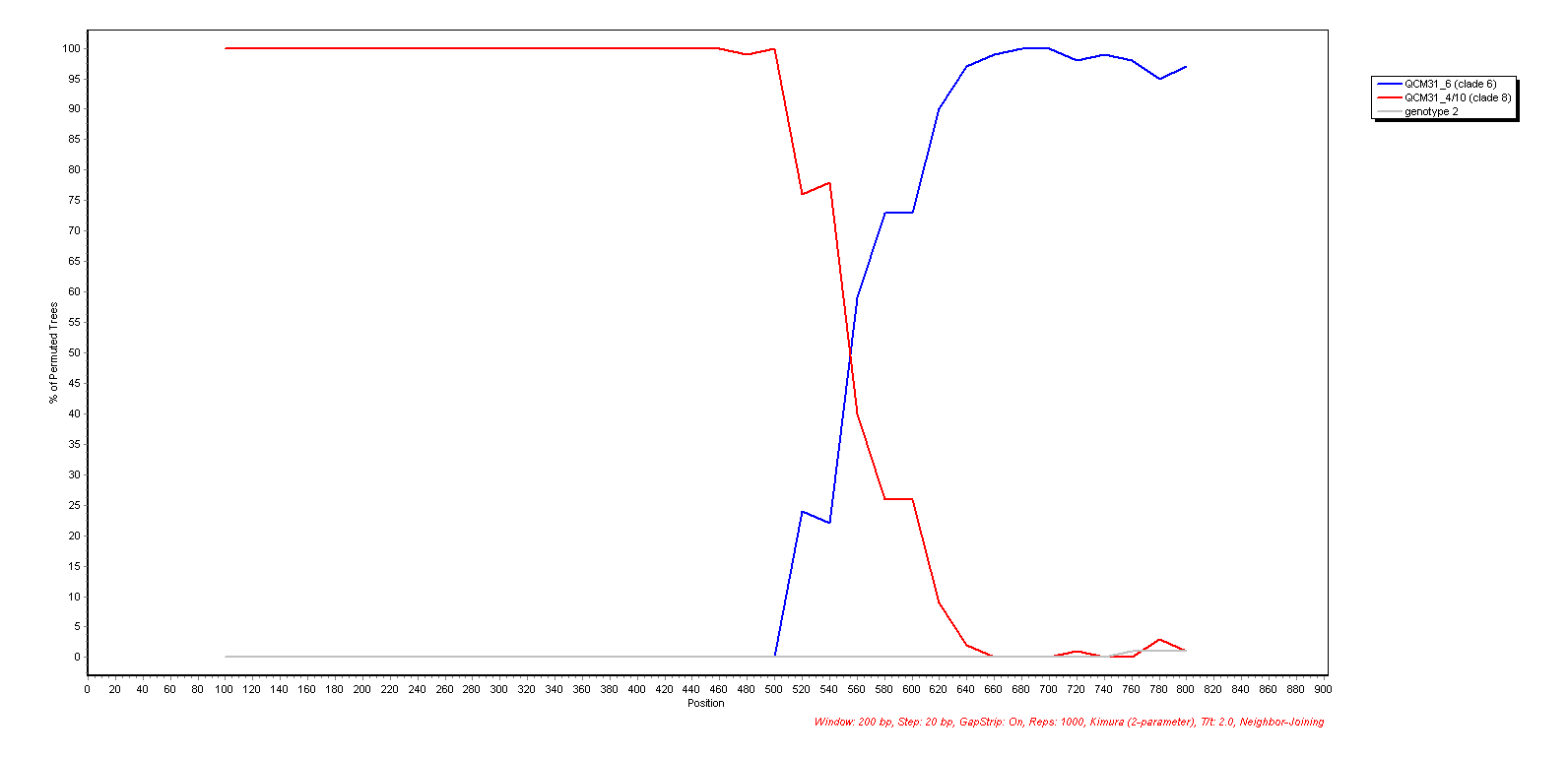

Supplement: S2 Fig — The bootscanning plot was carried out on each putative recombinant. The parameters used for analysis are shown on the bottom row of each figure. The QCM32_5 was not included in the bootscanning analysis for the unidentified minor parental sequences. (ZIP) [file pone.0161880.s002.zip › Supplemental Fig S2/QCM31_11.tif]

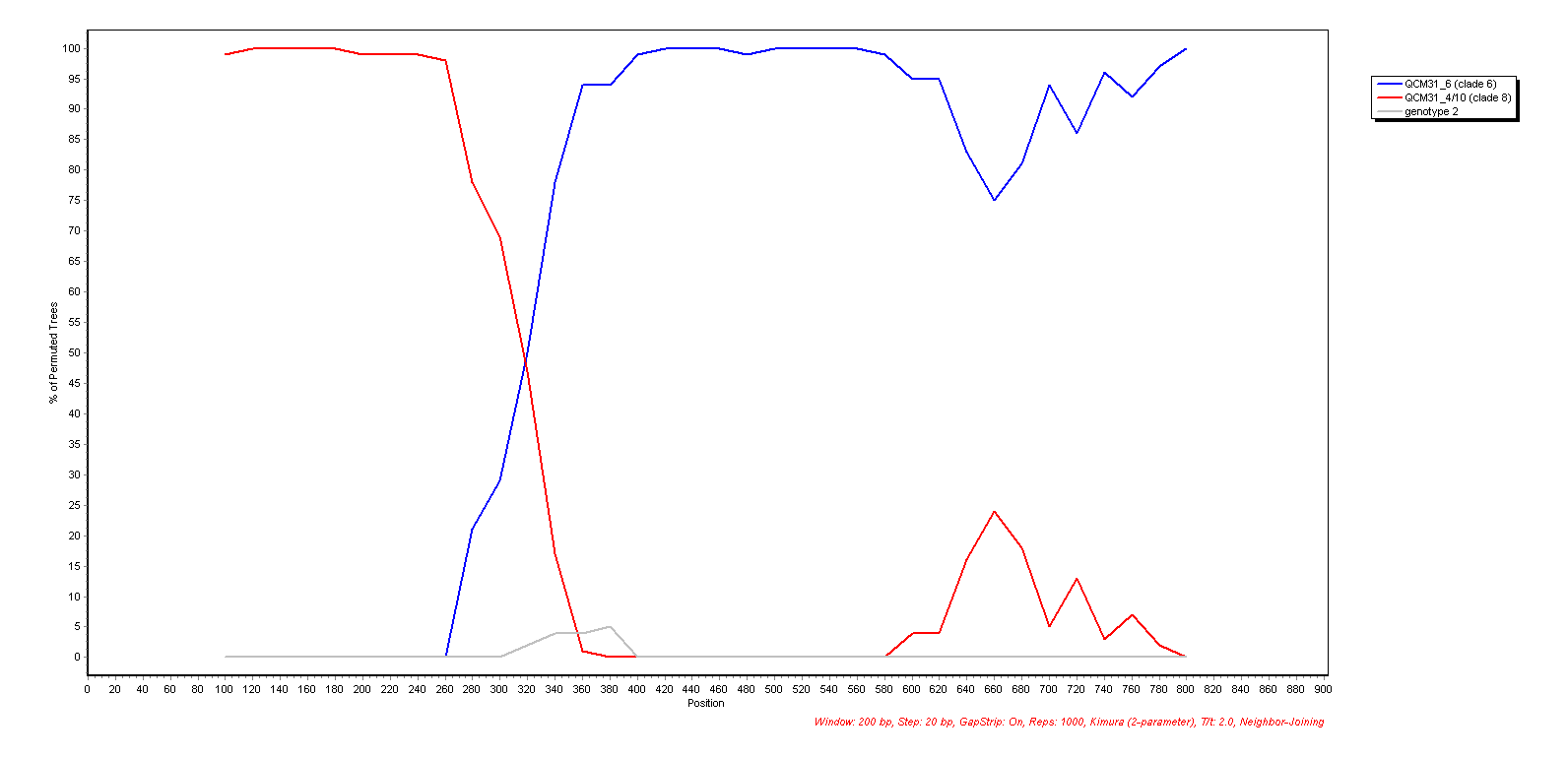

Supplement: S2 Fig — The bootscanning plot was carried out on each putative recombinant. The parameters used for analysis are shown on the bottom row of each figure. The QCM32_5 was not included in the bootscanning analysis for the unidentified minor parental sequences. (ZIP) [file pone.0161880.s002.zip › Supplemental Fig S2/QCM31_2.tif]

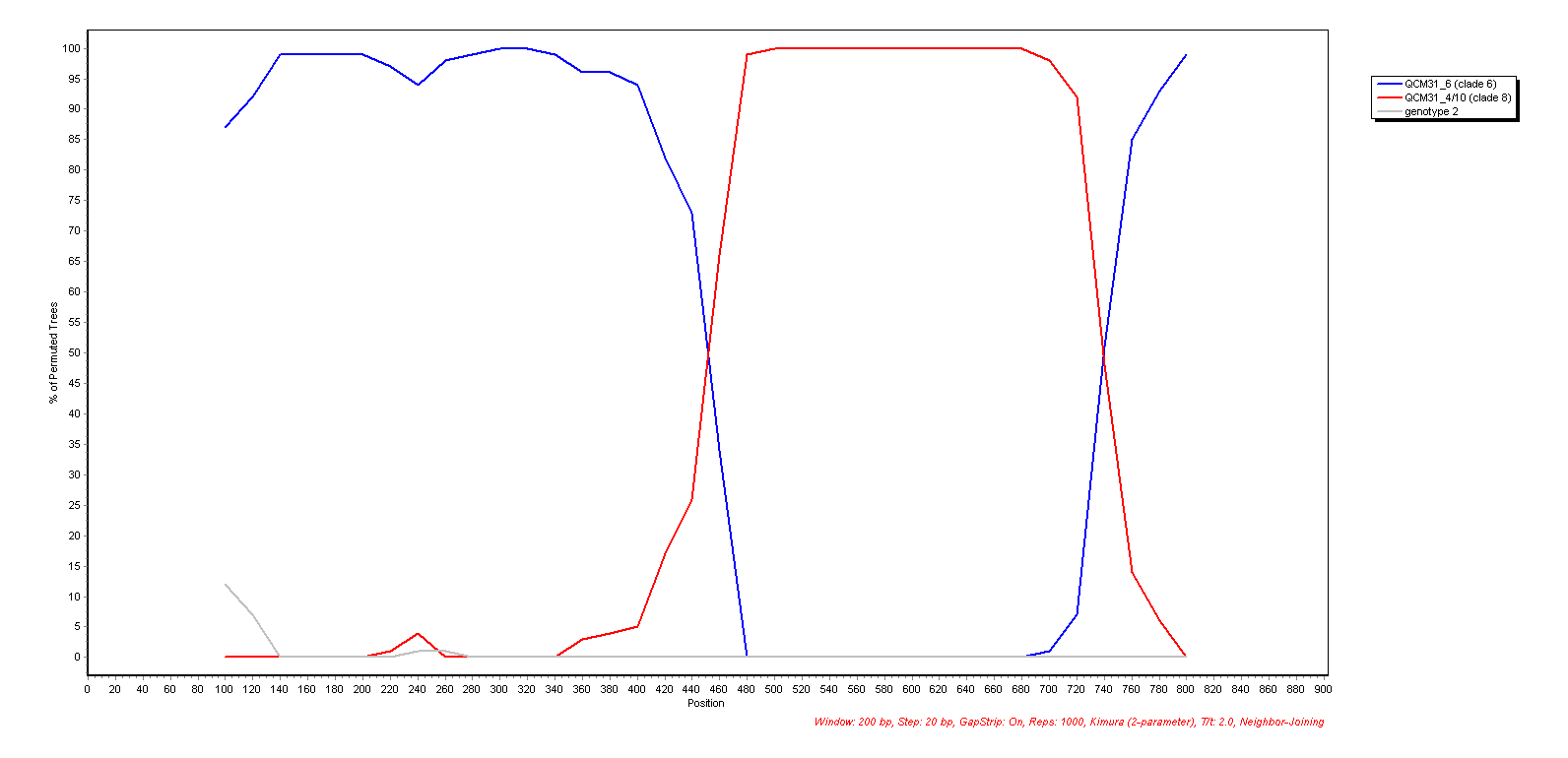

Supplement: S2 Fig — The bootscanning plot was carried out on each putative recombinant. The parameters used for analysis are shown on the bottom row of each figure. The QCM32_5 was not included in the bootscanning analysis for the unidentified minor parental sequences. (ZIP) [file pone.0161880.s002.zip › Supplemental Fig S2/QCM31_3.tif]

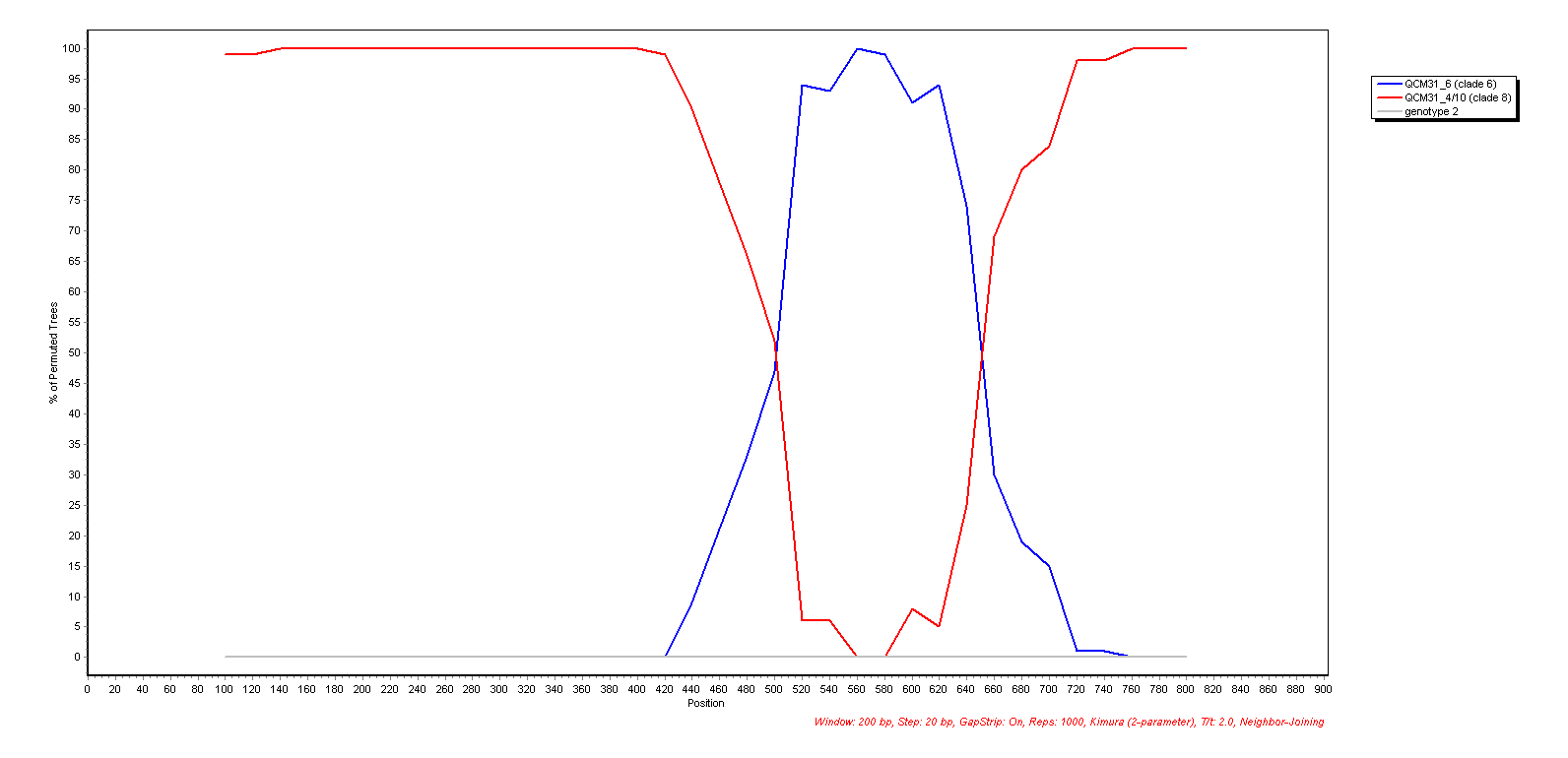

Supplement: S2 Fig — The bootscanning plot was carried out on each putative recombinant. The parameters used for analysis are shown on the bottom row of each figure. The QCM32_5 was not included in the bootscanning analysis for the unidentified minor parental sequences. (ZIP) [file pone.0161880.s002.zip › Supplemental Fig S2/QCM31_7.tif]

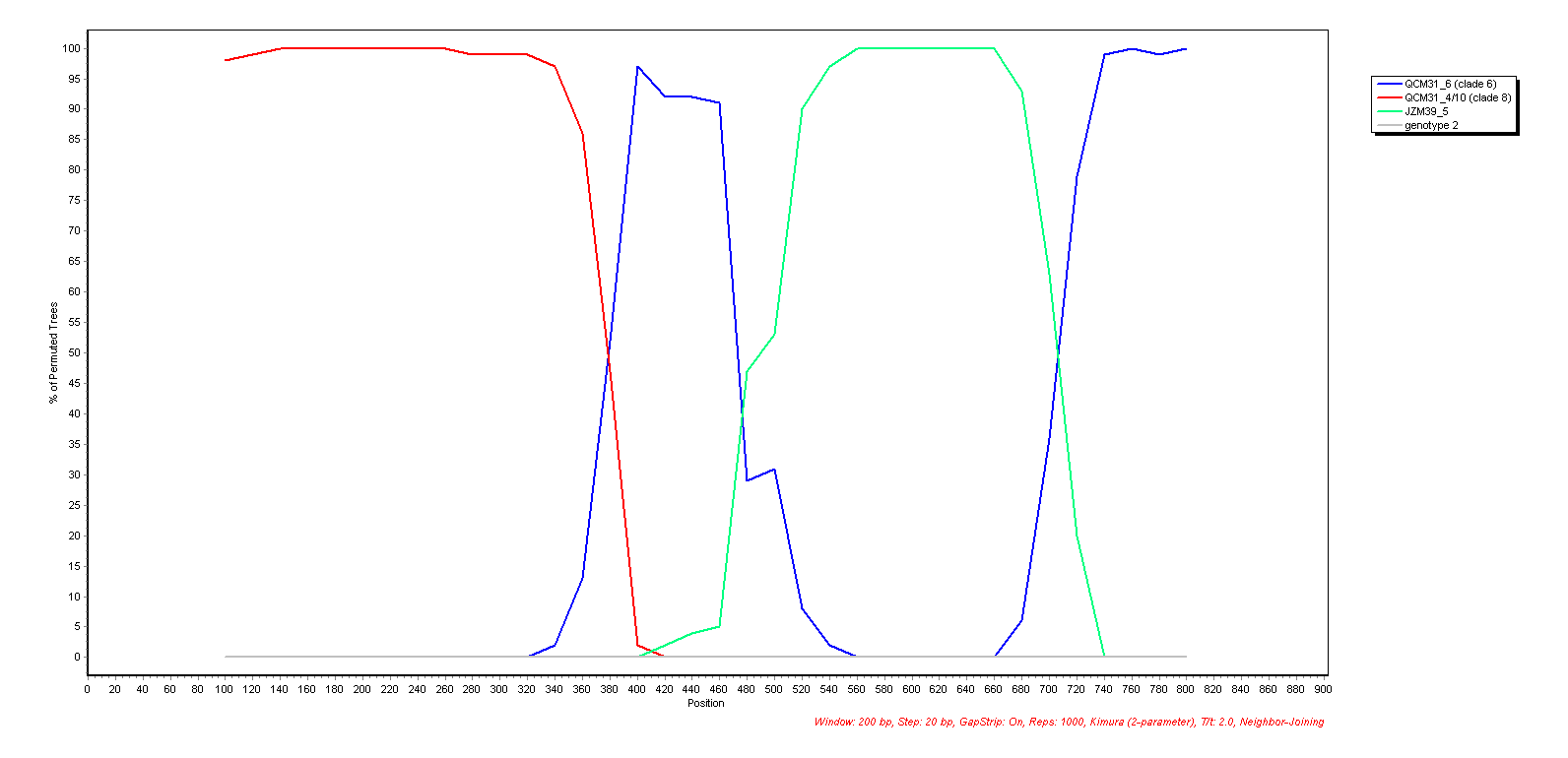

Supplement: S2 Fig — The bootscanning plot was carried out on each putative recombinant. The parameters used for analysis are shown on the bottom row of each figure. The QCM32_5 was not included in the bootscanning analysis for the unidentified minor parental sequences. (ZIP) [file pone.0161880.s002.zip › Supplemental Fig S2/QCM31_8.tif]

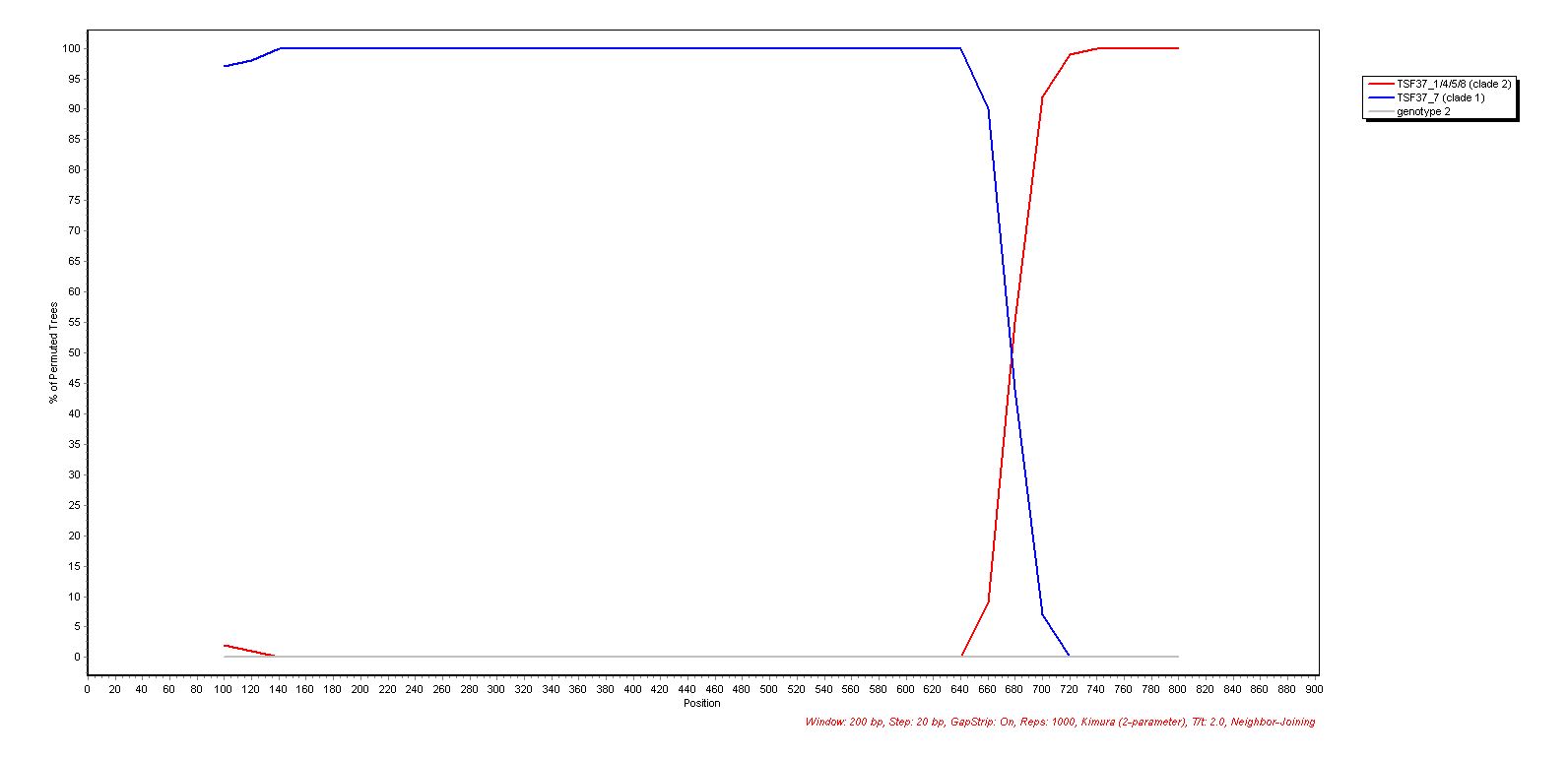

Supplement: S2 Fig — The bootscanning plot was carried out on each putative recombinant. The parameters used for analysis are shown on the bottom row of each figure. The QCM32_5 was not included in the bootscanning analysis for the unidentified minor parental sequences. (ZIP) [file pone.0161880.s002.zip › Supplemental Fig S2/TSF37_2+10.tif]

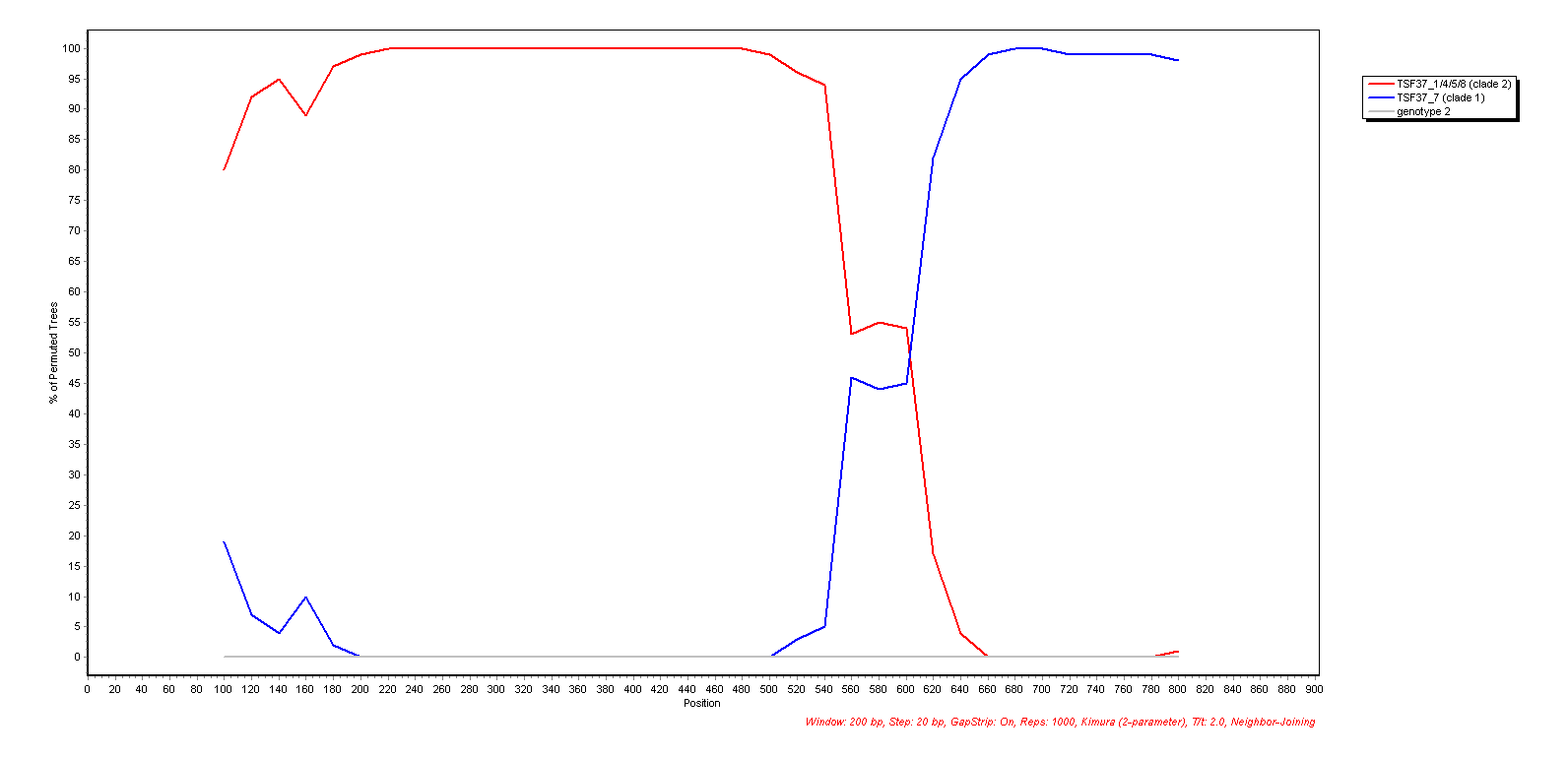

Supplement: S2 Fig — The bootscanning plot was carried out on each putative recombinant. The parameters used for analysis are shown on the bottom row of each figure. The QCM32_5 was not included in the bootscanning analysis for the unidentified minor parental sequences. (ZIP) [file pone.0161880.s002.zip › Supplemental Fig S2/TSF37_3.tif]

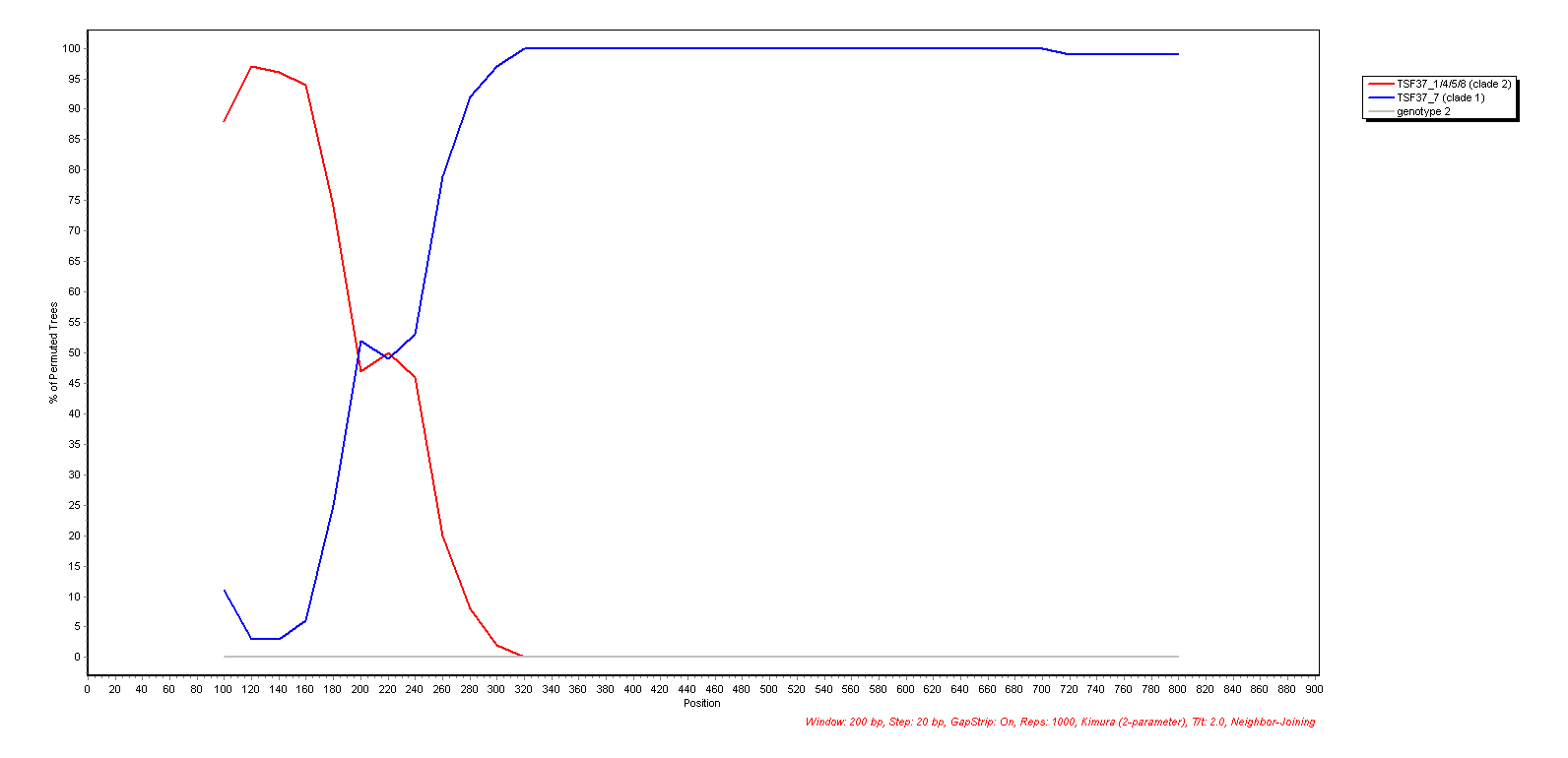

Supplement: S2 Fig — The bootscanning plot was carried out on each putative recombinant. The parameters used for analysis are shown on the bottom row of each figure. The QCM32_5 was not included in the bootscanning analysis for the unidentified minor parental sequences. (ZIP) [file pone.0161880.s002.zip › Supplemental Fig S2/TSF37_6.tif]

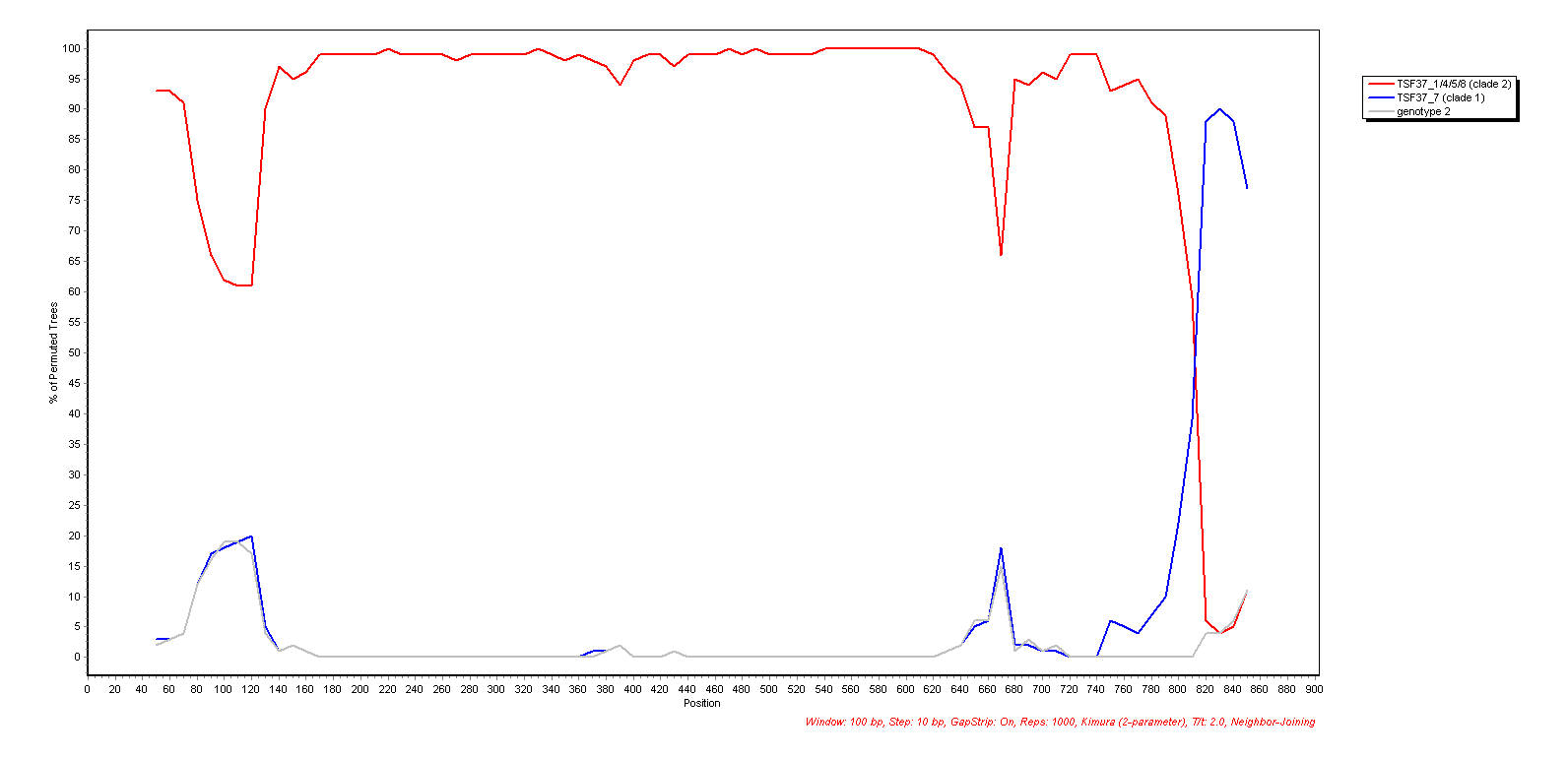

Supplement: S2 Fig — The bootscanning plot was carried out on each putative recombinant. The parameters used for analysis are shown on the bottom row of each figure. The QCM32_5 was not included in the bootscanning analysis for the unidentified minor parental sequences. (ZIP) [file pone.0161880.s002.zip › Supplemental Fig S2/TSF37_9.tif]

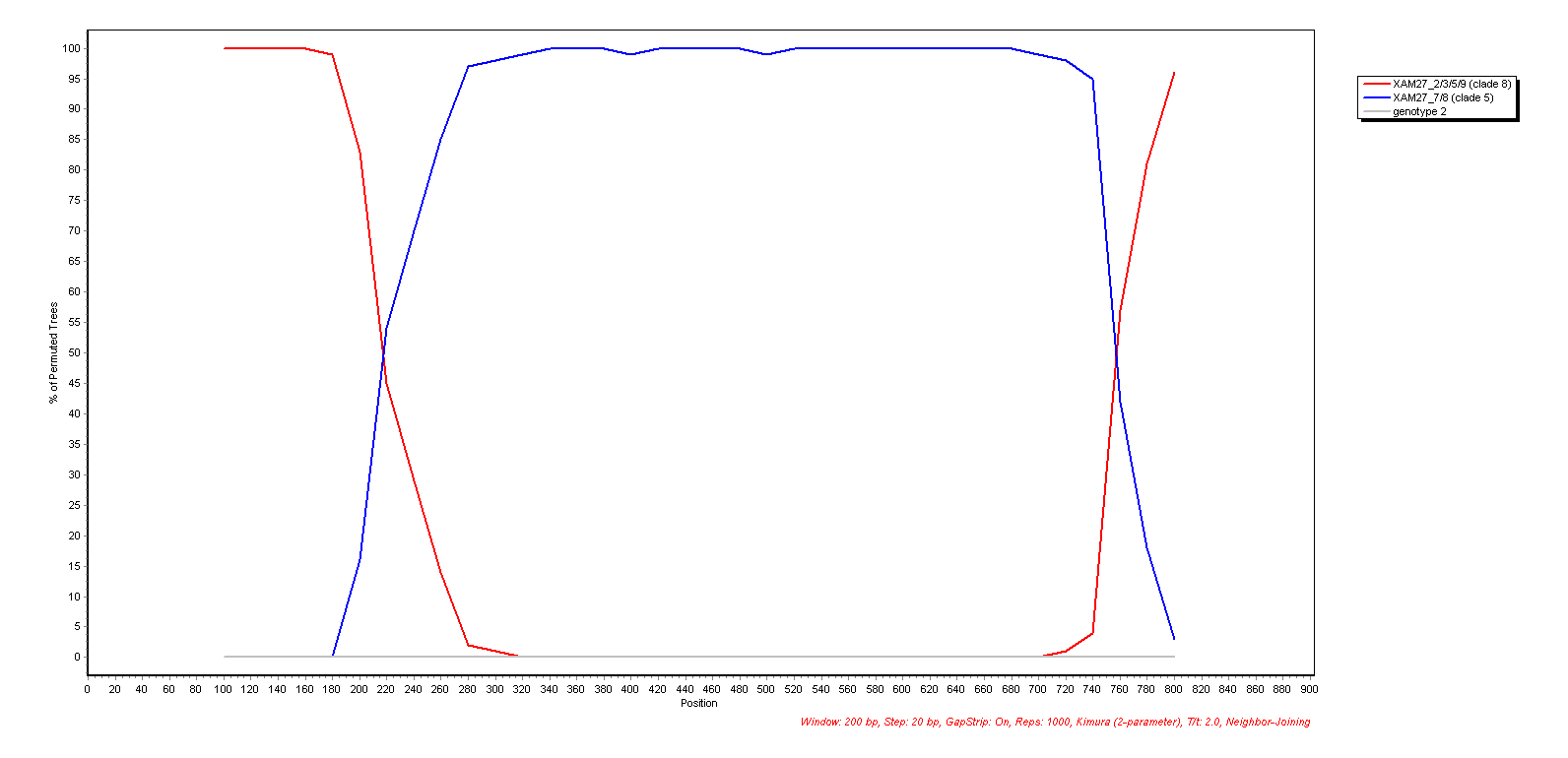

Supplement: S2 Fig — The bootscanning plot was carried out on each putative recombinant. The parameters used for analysis are shown on the bottom row of each figure. The QCM32_5 was not included in the bootscanning analysis for the unidentified minor parental sequences. (ZIP) [file pone.0161880.s002.zip › Supplemental Fig S2/XAM27_1.tif]

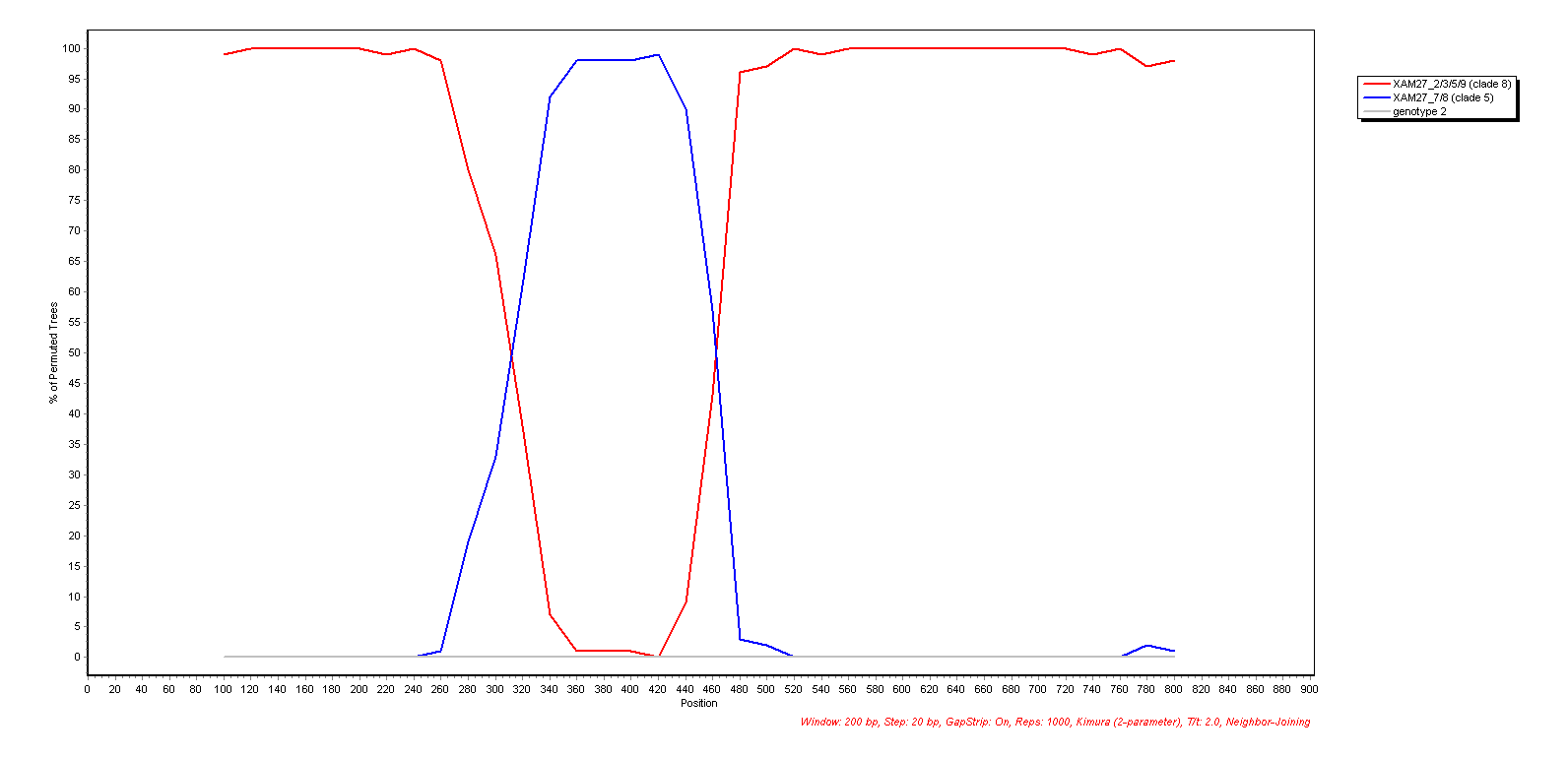

Supplement: S2 Fig — The bootscanning plot was carried out on each putative recombinant. The parameters used for analysis are shown on the bottom row of each figure. The QCM32_5 was not included in the bootscanning analysis for the unidentified minor parental sequences. (ZIP) [file pone.0161880.s002.zip › Supplemental Fig S2/XAM27_10.tif]

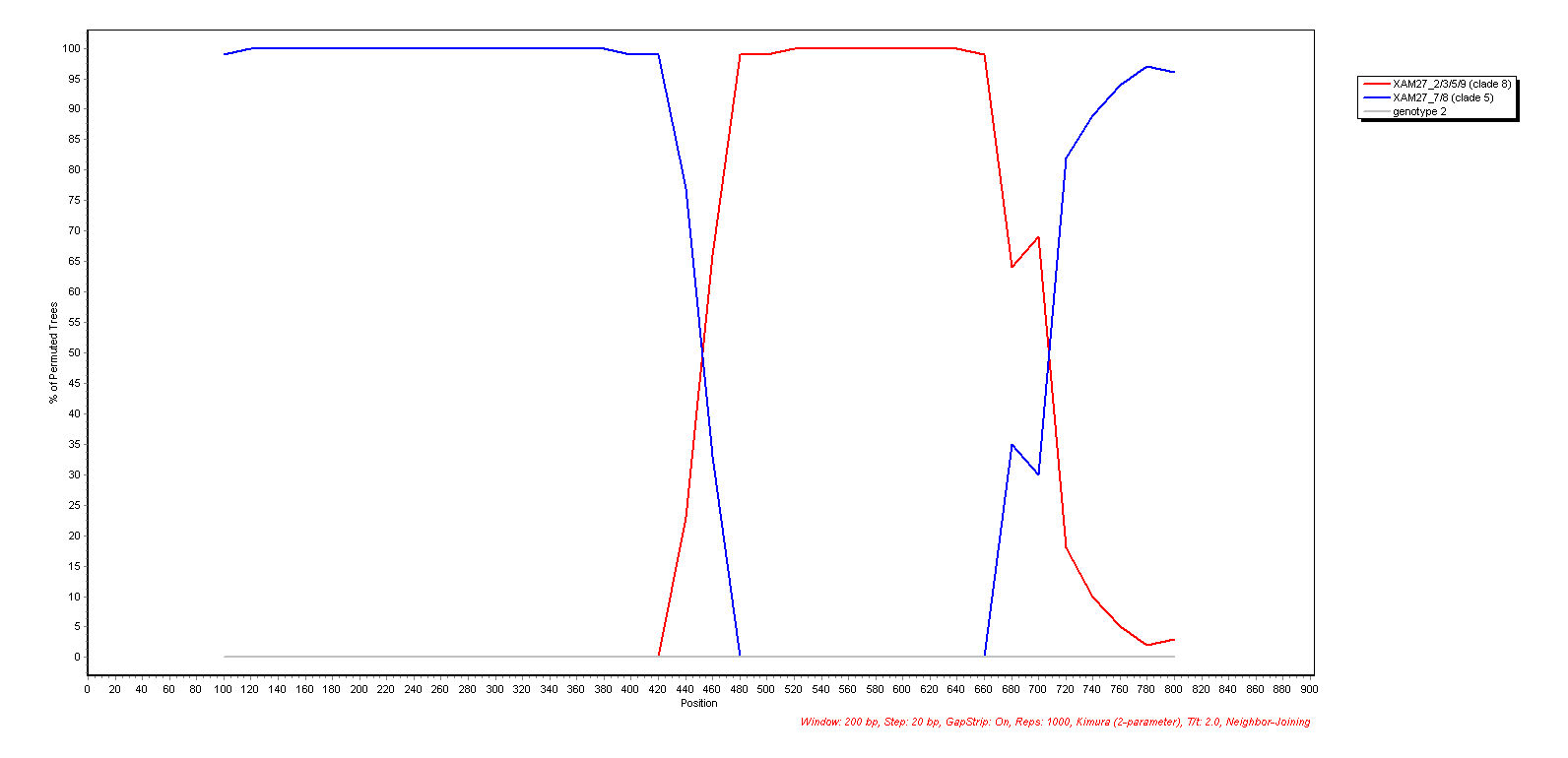

Supplement: S2 Fig — The bootscanning plot was carried out on each putative recombinant. The parameters used for analysis are shown on the bottom row of each figure. The QCM32_5 was not included in the bootscanning analysis for the unidentified minor parental sequences. (ZIP) [file pone.0161880.s002.zip › Supplemental Fig S2/XAM27_4.tif]

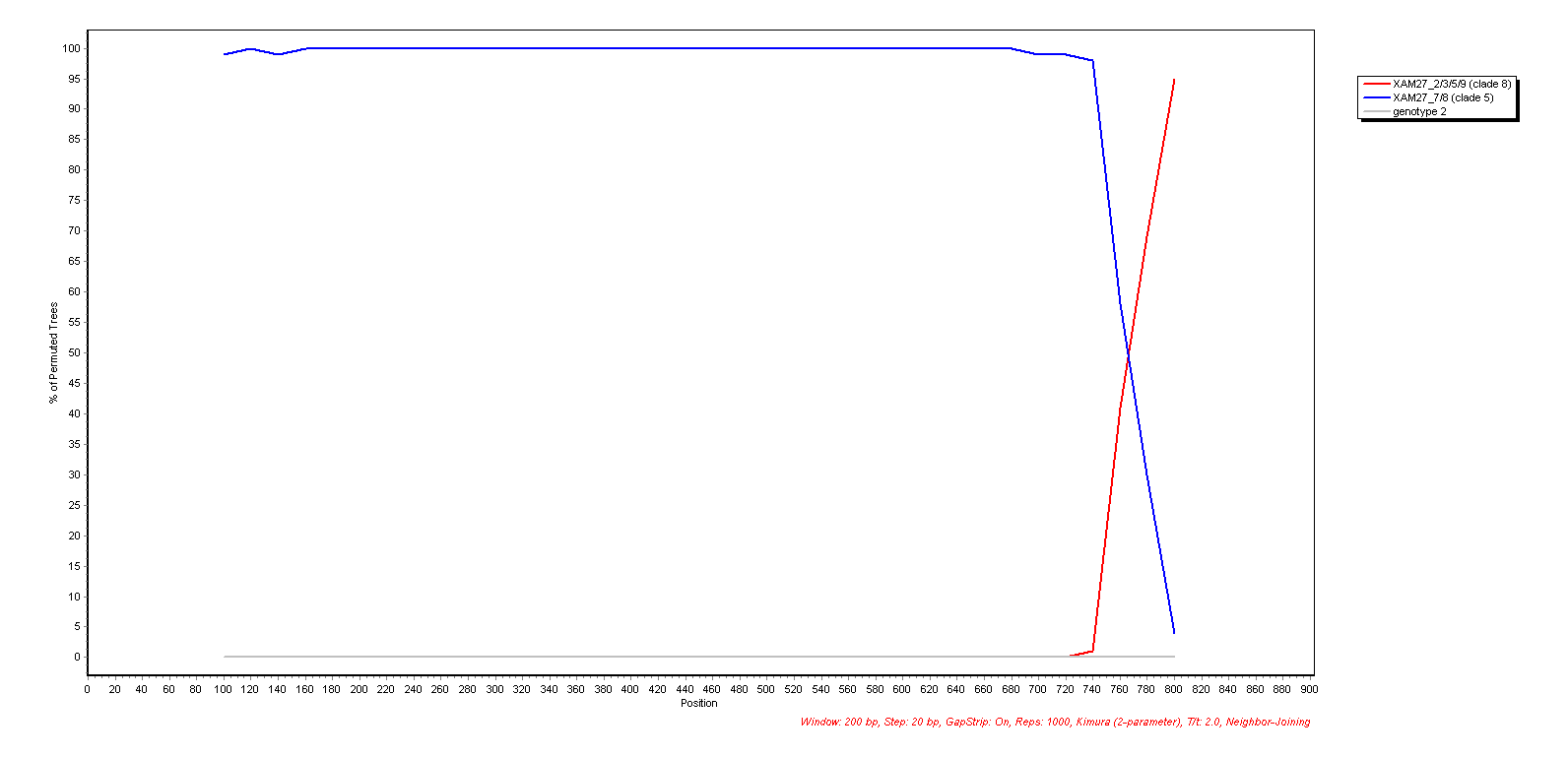

Supplement: S2 Fig — The bootscanning plot was carried out on each putative recombinant. The parameters used for analysis are shown on the bottom row of each figure. The QCM32_5 was not included in the bootscanning analysis for the unidentified minor parental sequences. (ZIP) [file pone.0161880.s002.zip › Supplemental Fig S2/XAM27_6.tif]
